# Immunization with Cocktail of HIV-Derived Peptides in Montanide ISA-51 Is Immunogenic, but Causes Sterile Abscesses and Unacceptable Reactogenicity

**DOI:** 10.1371/journal.pone.0011995

**Published:** 2010-08-10

**Authors:** Barney S. Graham, M. Juliana McElrath, Michael C. Keefer, Kyle Rybczyk, David Berger, Kent J. Weinhold, Janet Ottinger, Guido Ferarri, David C. Montefiori, Don Stablein, Carol Smith, Richard Ginsberg, John Eldridge, Ann Duerr, Pat Fast, Barton F. Haynes, the AIDS Vaccine Evaluation Group

**Affiliations:** 1 Vaccine Research Center, National Institute of Allergy and Infectious Diseases, National Institutes of Health, Bethesda, Maryland, United States of America; 2 Fred Hutchinson Cancer Research Center, University of Washington at Seattle, Seattle, Washington, United States of America; 3 University of Rochester School of Medicine and Dentistry, Rochester, New York, United States of America; 4 Vanderbilt University School of Medicine, Nashville, Tennessee, United States of America; 5 Duke University School of Medicine, Durham, North Carolina, United States of America; 6 EMMES Corporation, Rockville, Maryland, United States of America; 7 Profectus Biosciences, Inc., Tarrytown, New York, United States of America; 8 International AIDS Vaccine Initiative, New York, New York, United States of America; University of California San Francisco, United States of America

## Abstract

**Background:**

A peptide vaccine was produced containing B and T cell epitopes from the V3 and C4 Envelope domains of 4 subtype B HIV-1 isolates (MN, RF, CanO, & Ev91). The peptide mixture was formulated as an emulsion in incomplete Freund's adjuvant (IFA).

**Methods:**

Low-risk, healthy adult subjects were enrolled in a randomized, placebo-controlled dose-escalation study, and selected using criteria specifying that 50% in each study group would be HLA-B7+. Immunizations were scheduled at 0, 1, and 6 months using a total peptide dose of 1 or 4 mg. Adaptive immune responses in16 vaccine recipients and two placebo recipients after the 2nd immunization were evaluated using neutralization assays of sera, as well as ELISpot and ICS assays of cryopreserved PBMCs to assess CD4 and CD8 T-cell responses. In addition, ^51^Cr release assays were performed on fresh PBMCs following 14-day stimulation with individual vaccine peptide antigens.

**Results:**

24 subjects were enrolled; 18 completed 2 injections. The study was prematurely terminated because 4 vaccinees developed prolonged pain and sterile abscess formation at the injection site-2 after dose 1, and 2 after dose 2. Two other subjects experienced severe systemic reactions consisting of headache, chills, nausea, and myalgia. Both reactions occurred after the second 4 mg dose. The immunogenicity assessments showed that 6/8 vaccinees at each dose level had detectable MN-specific neutralizing (NT) activity, and 2/7 HLA-B7+ vaccinees had classical CD8 CTL activity detected. However, using both ELISpot and ICS, 8/16 vaccinees (5/7 HLA-B7+) and 0/2 controls had detectable vaccine-specific CD8 T-cell responses. Subjects with moderate or severe systemic or local reactions tended to have more frequent T cell responses and higher antibody responses than those with mild or no reactions.

**Conclusions:**

The severity of local responses related to the formulation of these four peptides in IFA is clinically unacceptable for continued development. Both HIV-specific antibody and T cell responses were induced and the magnitude of response correlated with the severity of local and systemic reactions. If potent adjuvants are necessary for subunit vaccines to induce broad and durable immune responses, careful, incremental clinical evaluation is warranted to minimize the risk of adverse events.

**Trial Registration:**

ClinicalTrials.gov NCT00000886

## Introduction

Development of an effective vaccine for HIV-1 remains a public health priority, and a recent report of partial efficacy suggests that it may be possible [1]. The Phase III trial in Thailand evaluated a recombinant canarypox vector expressing Envelope, Gag, and parts of Pol and Nef proteins from HIV-1 subtype B/E in combination with a recombinant HIV-1 B/E gp120 formulated in alum [1]. Although the mechanism of protection is currently unknown, the results of this study and experience gained from other successful viral vaccine development efforts suggest that both antibody and T cell responses will be important for preventing or controlling HIV-1 infection.

In this study, a polyvalent synthetic peptide was evaluated in healthy adults. It was designed to stimulate CD4 T-cells against a conserved region of the HIV-1 Envelope glycoprotein and to elicit both antibody and CD8 T-cell responses to the V3 loop region. The vaccine included peptide sequences from 4 different HIV-1 clade B variants (MN, Can0A, RF, and EV91) (Table 1), and was formulated with incomplete Freund's adjuvant (IFA). Montanide ISA-51 is a water-in-oil emulsion composed of mineral oil mixed with the surfactant mannose mono-oleate in a 1∶1 ratio with the aqueous phase. The primary study objective was to assess safety, and secondary objectives involved immunogenicity assessment of both humoral and cellular responses. Preclinical studies with this peptide formulation in mice and nonhuman primates demonstrated immunogenicity, including high titer antibody responses to V3, lymphoproliferative responses indicative of CD4 T-cell responses, and CD8 T-cell responses [2], [3], and did not demonstrate significant toxicity or local reactogenicity.

**Table 1:** Subject demographics.

	Control (N = 3)	1 mg (N = 12)	4 mg (N = 9)	Total (N = 24)
**Gender**				
***Female***	1	4	4	9
*Male*	2	8	5	15
**HLA**				
*B7*	1	6	3	10
*Other*	2	6	6	14
**Race/Ethnicity**				
*White*, *non-Hispanic*	2	12	7	21
*Black*, *non-Hispanic*	0	0	1	1
*Asian/Pacific Islander*	1	0	1	2
**Age**				
*Median*	25	33	44	33
*Range*	18–29	18–44	21–58	18–58
**Received Vaccine**				
*Day 0*	3	9	12	24
*Day 30*	2	8	8	18

Other peptide-based vaccines for HIV have achieved variable immunogenicity ranging from virtually no detectable responses to an orally administered octameric HIV-1 V3 peptide in alum [4], to consistent antibody and T cell responses detected in subjects immunized with lipopeptide-conjugated peptides [5], to intermediate responses in subjected immunized parenterally repeatedly with the octameric V3 peptide [6]. These peptide-based vaccines all were described as having acceptable local toxicity.

Although vaccines formulated with mineral oil have been administered to more than 1 million people since 1945, with the emergence of aluminum-based adjuvants, they fell out of favor because of the reactogenicity profile and potential for causing sterile abscesses [7]. With improvements in recent generations of IFA products that have more specific surfactants and refined oils that reduce the frequency of local and systemic adverse events, the use of these potent water-in-oil adjuvant formulations was adopted for immunotherapeutic vaccines for fatal diseases including HIV-1 infection. Two peptide-based vaccines, including the one being evaluated in the current study, formulated with Montanide ISA-51 have previously been evaluated in a total of 18 HIV-infected subjects as candidate therapeutic vaccines [8], [9], and judged to be safe. The Montanide ISA-51 adjuvant was also evaluated in other therapeutic vaccine trials in more than 100 HIV-infected subjects, formulated with whole inactivated virus and was judged to be safe [10], [11]. Montanide ISA-51 has also been frequently used as an adjuvant in a variety of therapeutic cancer vaccines in which reactogenicity is never described as greater than moderate or Grade 2. Therefore, in human subjects who have underlying conditions that may affect immune competence, reactogenicity does not appear to be a limiting factor.

In healthy adults without underlying conditions there is a mixed history of reactogenicity from IFA, which has been extensively reviewed, with the conclusion that for serious infections like malaria and HIV, the use of IFA may be justified [12]. Since the current study was undertaken, another trial has been conducted in healthy adults evaluating safety and immunogenicity of Montanide ISA-51 formulated with a candidate malaria vaccine comprised of Pfs25 and Psv25 surface proteins [13]. This malaria study team had been informed about the results of the current study. This trial was prematurely terminated due to unexpected systemic reactogenicity. Six subjects experienced severe local reactions involving swelling and induration at the injection site, which were not unexpected based on the results of the current study. In 5 of the 6 subjects, the reaction subsided within 8 weeks, and in the other it resolved in about 6 months. Unexpectedly, in the malaria vaccine study, 2 out of 30 vaccine recipients developed erythema nodosum and 2 others experienced leukemoid reactions [13]. We now report our experience with Montanide ISA-51 IFA formulated with HIV peptide antigens, to inform future investigations of this adjuvant in healthy adult volunteers.

## Methods

### Ethics Statement

These studies were approved by the Institutional Review Boards (IRB) at each clinical site (Vanderbilt University IRB, University of Rochester IRB, and Fred Hutchinson Cancer Research Center IRB), and were performed in accordance with 45 CFR Part 46, U.S. Food and Drug Administration regulations, and principles expressed in the Declaration of Helsinki. All subjects signed written informed consent documents.

### Objectives

The purpose of the study was to evaluate the safety and immunogenicity of a polyvalent HIV-1 C4-V3 synthetic peptide mixture formulated in Incomplete Freund's Adjuvant (IFA, mineral oil containing mannose mono-oleate). Four synthetic peptides based on the HIV-1 clade B strains MN, EV91, RF, and CANO are included in the candidate vaccine. Each of these four component peptides consists of two sections or parts. The first, the “C4” section, is a 15 amino acid sequence corresponding to a potent activator of anti-HIV memory helper T cells present in the fourth conserved region of HIV-1 gp120. The second part, the “V3” section, is a 24 amino acid sequence which corresponds to the V3 loop region of gp120 of the particular HIV-1 strain. The V3 region is an established B cell epitope recognized by anti-HIV neutralizing antibodies, as well as being an HLA-B7 restricted CTL epitope. The same “C4” sequence 15 is used in all four of the pooled fusion peptides, while the V3 portion of the fusion peptide is unique for each strain. The trial was sized to achieve the primary objective which was to evaluate the safety of the C4-V3 peptides formulated in IFA in HIV-1 uninfected volunteers, and secondary objectives were to evaluate the humoral and cellular immune responses. The protocol for this trial and supporting CONSORT checklist are available as supporting information; see Checklist S1, Diagram S1, Protocol S1.

### Study design

Healthy adult volunteers between 18 and 60 years of age were eligible for enrollment. The study was randomized, placebo-controlled and double-blind. Statisticians assigned the randomization sequence to the pharmacy using a random number generator. Clinicians, subjects, and laboratory investigators were all blinded to the assignments. The goal was to enroll 28 subjects, half of whom had the HLA-B7 allele known to present the V3 epitope contained in the vaccine [14]. 12 subjects would receive a total of 1 mg peptide (250 µg of each); 12 would receive 4 mg peptide (1 mg of each); and 4 would receive the placebo preparation which was adjuvant alone without peptide. Subjects were scheduled to receive 4 injections at 0, 1, 6, and 12 months. Subjects were injected with 0.5 ml in both arms. After each immunization, subjects were observed for 30 minutes and monitored for temperature and local reactions. Subjects recorded their own temperature daily for 3 days and recorded any signs and symptoms. Each subject was seen on either day 1 or 2 post vaccine in the clinic and contacted by phone on day 7 to review the symptoms during the immediate post-vaccination period. Laboratory evaluations including urinalysis, alanine transaminase (ALT), gamma-glutamyl transpeptidase (GGT), creatinine, CBC with differential counts and platelets, and CD4/CD8 T cell counts were assessed prevaccination, 4 weeks following the first vaccination, and 2, 4 and 24 weeks following the second vaccination. Subjects were originally scheduled to be followed for an additional 6 months following the month 12 injection. DTH skin testing was done in a subset of subjects at day 378. See Protocol S1 for additional details including specific inclusion and exclusion criteria (Pages 13–14).

The study was approved by the U.S. Food and Drug Administration and registered with ClinicalTrials.gov (#NCT00000886). The local Institutional Review Board of each participating site approved the study, and a Data and Safety Monitoring Committee provided oversight, in addition to the site clinicians and the protocol safety team that included a Division of AIDS medical monitor.

### Vaccine Preparation and Delivery

The four peptide sequences are shown in Table S1. Peptide immunogens were manufactured and quality controlled by a commercial vendor for FDA IND submission and approval. Peptides were mixed in equal concentrations and combined with Montanide ISA-51 using the Kirkland EmulsiFlex 1000™ device. The emulsion stability was confirmed with the “water-drop” test. This test was performed in a 60×15 mm petri dish containing 4°C water. After preparing the sterile syringe for vaccine administration, one drop of the remaining emulsion was dropped from 1 cm above the water with the syringe held at a 60° angle. To meet the criteria for a stable emulsion, the drop had to remain intact on the water surface for 3 minutes. If the emulsion was acceptable, it was delivered as a 0.5 ml injection into each deltoid muscle. For additional details see Appendix D in the protocol posted in Protocol S1 online. The placebo control was Montanide ISA-51without peptide.

### Antibody responses

Neutralizing antibodies were assessed against HIV-1_MN_ in an MT-2 cell-killing assay as described [15]. Briefly, 500 TCID_50_ of virus were incubated with multiple dilutions of heat-inactivated serum samples in triplicate for 1 hr at 37°C in 96-well flat-bottom culture plates. Cells (5×10^4^) in 100 µl of growth medium were added and the incubation continued until most but not all of the cells in virus control wells (cells+virus but no serum sample) were involved in syncytium formation (usually 3-5 days). Cell viability was quantified by neutral red uptake [15]. Neutralization titers were defined as the reciprocal serum dilution (before the addition of cells) at which 50% of cells are protected from virus induced killing. Each set of assays included a positive control serum that had been assayed multiple times and had a known average titer. The assay stock of HIV-1_MN_ was grown in H9 cells and titrated in MT-2 cells.

### T cell responses

The measurements of CD8+ T cell function were performed by the AVEG Central Laboratory at Duke University. The lytic assay was performed with fresh PBMCs using a standard 4-hour ^51^Cr-release assay. Effectors were stimulated with peptides for 14 days and targets were peptide-labeled autologous BLCL. IFN-γ ELISpot assays were performed after 16-hour peptide stimulation of 2×10^5^ cryopreserved PBMCs. Positive responses were defined as a 3-fold response above background and >2-fold reduction following depletion of CD8+ T cells as described [16]. Intracellular IFN-γ production was analyzed by flow cytometry after 6-hour peptide stimulation. CD33+CD62P expression was used to exclude monocytes and activated platelets, and final analysis plots of IFN-γ-APC vs. CD69-PE were gated on CD4^+^ or CD8^+^ lymphocytes. Background gates were set at 0.05%, and positive results were defined as >2-fold background.

### Statistical analysis

Descriptive summaries with frequencies of reactogenicity measures, immune assay response rates and geometric mean titer results are presented along with nonparametric comparisons of antibody levels and reaction severity. An exact 2-sided 95% confidence interval for the rate of sterile abscess or delayed local reaction is calculated.

## Results

Twenty-four of the projected 28 study participants were enrolled at three sites (Rochester, NY, Nashville, TN and Seattle, WA) between 10/13/1997 and 03/05/1998. There were 15 male and 9 female participants; 21 were White, 1 was Black and 3 were Asian/Pacific Islanders. The median participant age was 33 years (range 18–58). Four participants had the HLA A2 haplotype and 10 had HLA B7. All 24 participants received the first study vaccination; 18 received the second. Two participants withdrew after the first vaccination and were lost to follow-up; 4 participants did not receive the second vaccination due to discontinuation of vaccination as described below (Table 1).

### Local reactogenicity

**Figure 1:**
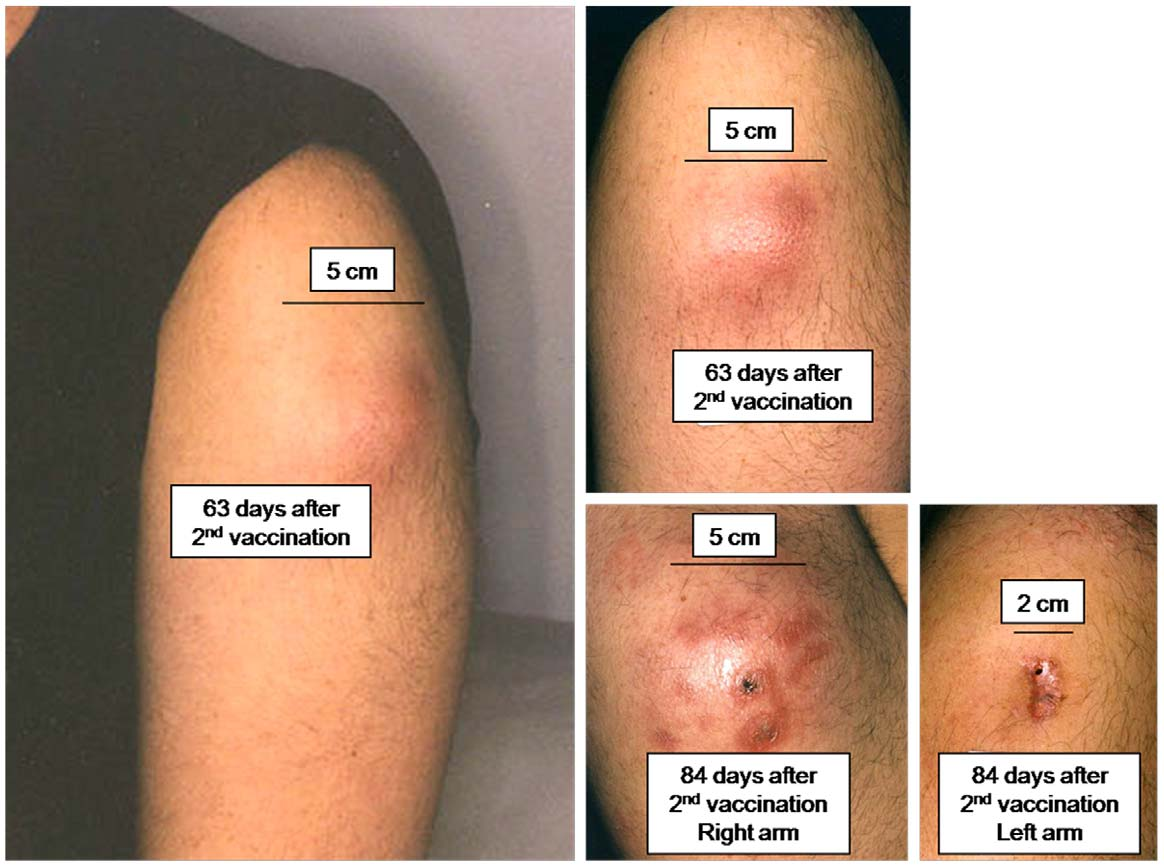
Case report of local reaction and sterile abscess formation. Two days after the 1^st^ immunization, a 22 year old man in the 1 mg dose cohort developed mild tenderness over the injection site that resolved on day 4. After the 2^nd^ immunization was administered 28 days later, mild tenderness occurred that lasted for one day. 15 days after the 2^nd^ immunization a 0.25 cm asymptomatic subcutaneous nodule was detected on routine physical exam. There was no erythema at that time. Fifty days post 2^nd^ immunization the volunteer reported the onset of throbbing pain and that was treated with warm compresses and nonsteroidal anti-inflammatory drugs. On day 56 there was erythema and induration of 8 and 9 cm diameter over the prior injection sites, and the subject was afebrile with a WBC of 9,600/cm^2^. On day 63 there was less pain and the area of induration was unchanged. However, the induration was less firm and the central area of erythema (A and B) was fluctuant. On day 84 there was spontaneous drainage from the lesions, from multiple fistulas on the right arm (C) and a single fistula on the left (D). The clinical course of all 4 subjects with sterile abscess formation is reviewed in Table S2.

Reactogenicity was evaluated in the immediate post-vaccination period by self-reported diary cards. Local reactogenicity assessments included pain, tenderness, induration, and erythema. During the first 7 days after each vaccination there was nothing unusual about the local reactions (Table 2). In four subjects (2 receiving 1 mg and 2 receiving 4 mg), painful swelling developed over both deltoids beginning between 1 week and 2 months after vaccination. The swelling included induration and central erythema, and subjects often woke from sleep because of the pain. In two subjects the painful swelling occurred after the first dose, and they did not receive the second vaccination. In both of these subjects the lesions resolved spontaneously without drainage. The other two developed the lesions after the second vaccination; one received the 1 mg dose and the other received the 4 mg dose. In these subjects, the induration, pain, and swelling increased until spontaneous drainage occurred between 10 and 11 weeks post-vaccination. The lesions over the right and left arms in one subject spontaneously drained within one day of each other. In the other subject, the spontaneous drainage in one arm was followed by aspiration and incision and drainage of the other arm 12 weeks later. Once drainage was established, the lesions gradually resolved and healed, in one case leaving a hyperpigmented scar (Figure 1, Table S2). These were all sterile abscesses, and no pathogenic organisms were isolated from any of the lesions or exudates. In summary, sterile abscesses were observed in 4/21 treated subjects, 19.1% (95% CI 6.8% to 39.8%), after which further vaccinations were terminated by joint recommendation of the study investigators and safety oversight committee.

**Table 2:** Local reactions* within 7 days of vaccination.

	Vaccination #1					Vaccination #2				
	N	None†	Mild	Moderate	Severe	N	None	Mild	Moderate	Severe
1 mg	12	1	9	2	0	8	1	6	1	0
4 mg	9	1	4	4	0	8	0	5	3	0
control	3	0	3	0	0	2	1	1	0	0

*Local reactions included: pain, tenderness, erythema, induration.

† None = no pain or tenderness; Mild = minimal pain or tenderness, no limitation of arm use; Moderate = notable pain or tenderness, some limitation of use of arm; Severe = extreme pain or tenderness, complete limitation of use of arm.

### Systemic reactogenicity

Systemic reactogenicity self-assessments were done for 3 days after each vaccination and included malaise, myalgia, headache, fever, chills, and nausea (Table 3). Severe systemic reactions began between 2 and 6 hours post-immunization. The most severe systemic reactions occurred in the 4 mg dose group and after the second immunization. The symptoms were characterized by sudden onset of headache, chills, nausea, and myalgia. The symptoms resolved in 24–48 hours. None of the subjects with the delayed sterile abscess formation had moderate or severe systemic reactogenicity. There were no significant or notable hematologic, renal, or hepatic abnormalities in any of the subjects at any time during the study.

**Table 3:** Systemic reactions* within 7 days of vaccination.

	Vaccination #1					Vaccination #2				
	N	None†	Mild	Moderate	Severe	N	None	Mild	Moderate	Severe
1 mg	12	6	4	2	0	8	3	2	3	0
4 mg	9	6	2	1	0	8	1	2	3	2
control	3	2	1	0	0	2	1	1	0	0

*Systemic reactions included: malaise, myalgia, headache, fever, nausea.

† None = no symptoms; Mild = symptoms require no change in activity or medication; Moderate = symptoms require modification of activity or medication; Severe = symptoms are incapacitating, requiring bed rest and/or loss of work or cancellation of social activities.

### Antibody response

All 20 vaccine recipients who remained in follow-up had detectable ELISA antibody responses to at least one peptide at day 196, and nine of 20 (45%) remained positive at day 378. None of the subjects had a detectable ELISA antibody response to gp160_IIIB_. The peak frequency of ELISA antibody to the MN V3 peptide was 13/20 (65%) at day 196. 75% of subjects had low level neutralizing activity against HIV-1_MN_ (Table 4). At day 56, ELISA antibody responses were assessed to V3 peptides from Can0, EV91, MN, and RF, and to full length gp160. The magnitude of the antibody response measured by OD tended to correlate with the severity of reactogenicity. Comparing subjects with moderate or severe (n = 10) local or systemic reactions to those with no reactions or mild (n = 11) yielded respective Wilcoxon test p-values of .07, .17, .06, and .06 for the Can0, EV91, MN, and RF V3 responses The association appeared to be related primarily to systemic reactions since the rank correlations were significant between systemic grade reactions for 3 of the V3 peptides and no trends toward correlation were observed for any antigen with the severity of local reactions.

**Table 4:** Antibody response on day 56 in subjects who received 2 doses.

		ELISA			50% neutralization assay		
		Frequency (mean O.D.)			Frequency and reciprocal serum dilution		
Group	N	C4-V3 – RF*	C4-V3 – MN	V3 MN	MN	Geometric mean titer	Median
IFA control	2	0/2	0/2	0/2	0/2	-	-
1 mg	8	8/8 (1.03)	7/8 (0.33)	4/8 (0.46)	6/8	42	29
4 mg	8	8/8 (1.62)	6/8 (0.69)	6/8 (0.44)	6/8	41	38

*C4-V3 refers to the entire peptide antigen used in the vaccine.

### T cell responses

One subject in each of the active dose groups had a detectable CD8 T-cell response measure by ^51^Cr-release at day 42, 14 days after the second immunization. Both subjects were HLA-B7+. Both of those subjects also had positive CD8 ELISpot responses. Those 2 subjects and 3 other HLA-B7+ vaccinees had positive CD8 ICS responses. Therefore, of those who received two immunizations, a total of 5/7 HLA-B7+ subjects had evidence of vaccine-induced CD8 T-cell responses, while only 3 of 9 HLA-B7-negative subjects had detectable CD8 T cell responses (Table 5). Six of the 8 CD8 T cell responders had moderate or severe systemic reactions to immunization or sterile abscess formation, while only 3/8 nonresponders had moderate systemic reactions and no sterile abscesses. DTH skin testing showed that 7/16 vaccinees had >5 mm reaction to 10 µg of peptide mixture. Neither of the two tested placebo recipients had positive responses. Response rates to Candida and tetanus antigens were 9/16 and 5/16, respectively, among vaccinees and 1/2 and 2/2 in placebo recipients.

**Table 5:** T cell responses in subjects who received 2 doses of vaccine.

		Frequency of Positive Responses at Day 42			
Group	N	^51^Cr CTL	CD8 ELISpot	CD8 ICS	CD4 ELISpot or ICS
IFA control	2	0	0	0	0
HLA-B7+	7	2	2	5	3
HLA-B7−	9	0	2	1	3

## Discussion

We report the Phase I clinical evaluation of a peptide-based candidate HIV vaccine formulated as an emulsion with Montanide ISA-51. The 4 peptides were designed to elicit CD4 T-cell responses to conserved regions in Envelope, and antibody and CD8 T-cell responses to epitopes contained in gp120 V3. To evaluate the vaccine induction of CD8 T-cell responses, 50% of the enrolled subjects were HLA-B7+ which is known to be the restriction element for the V3 (RPNNNTRKSI) CTL epitope. This product concept was extensively characterized and in preclinical studies was shown to elicit antibody and CD4 and CD8 T-cell responses [8], [17], [18], [19], [20], [21]. Although the product was shown to be immunogenic after two doses in this study, immunogenicity was not fully evaluated because the trial was stopped early due to unacceptable local reactogenicity. Therefore, this analysis is focused primarily on the safety endpoints of the trial.

The primary differences between the immunotherapy studies done with this product formulated in Montanide ISA-51 and the current studies are: 1) the immunological health of the subjects, 2) the 0.5 ml instead of 0.25 ml dose volume, and 3) the strength of the emulsion created with the EmulsiFlex 1000™ mechanical device. In preclinical toxicity studies, the clinical product had been tested in rabbits and rhesus monkeys. In one of two monkeys given a series of three immunizations with an 8 mg dose of peptide, granulomatous inflammation was noted at necropsy at the injection site. Otherwise, there was nothing notable about local toxicity.

The distinct differences in local reactogenicity between the current study and the studies evaluating therapeutic vaccines in HIV-infected subjects suggest that underlying host immune competence may be a primary determinant of the intensity of the local response to the Montanide ISA-51 adjuvant. This is supported by the prolonged local reactions in the Pfs25/Pvs25 malaria vaccine study in healthy subjects [13]. Even though sterile abscesses requiring drainage were not reported, there was induration lasting up to 6 months. It is also supported by the studies using this adjuvant with therapeutic vaccines for a variety of neoplasms in which the vaccines were characterized as well-tolerated [12], [22]. Two of the subjects in the present study with the delayed subcutaneous reactions and prolonged induration or abscess formation had reported arm soreness from overuse at the time of the initial vaccination (one from floor scrubbing and the other from skiing). The role of significant arm exercise in the subsequent reaction is unknown.

The assumption is that sterile abscess formation was related to the inflammatory effect induced by the adjuvant. Licensed aluminum-based adjuvants rarely are associated with sterile abscess formation, and the cause is thought to be related to aluminum hypersensitivity reactions [23]. The high frequency of local reactions in this study suggests adjuvant-induced inflammation rather than hypersensitivity was the cause of abscess formation. The adjuvant effect of IFA is in part related to the Toll-like receptor (TLR) stimulation by products contained in the oil, and in part by a depot effect. The removal of the vaccine dose in a stable emulsion by professional antigen presenting cells can take months. Therefore, using a higher dose volume would not only enlarge the central nidus of a sterile abscess, but would prolong the length of the inflammatory stimulus underlying the genesis of the abscess. The volume of the vaccine dose in the HIV therapeutic trial using this product was 0.25 ml. The dose volume in the Pfs25/Pvs25 malaria study was 0.5 ml. Based on the experience in this study with a 0.5 ml dose, we suggest that the lowest possible volume needed to deliver an adequate antigen dose should be used with potent adjuvants that have the potential for sterile abscess formation.

The method of producing the emulsion was the major difference between the current study and the trial done in healthy adults with malaria antigens in which there were some prolonged local reactions and two cases of erythema nodosum [13]. The emulsion was intentionally produced to be stable in the current study to reduce the potential for escape of the oils to organs like the spleen and liver, where they could potentially serve as a stimulus for granulomatous inflammation. One could speculate that the erythema nodosum seen in the malaria trial was related to a slightly less stable emulsion, and the sterile abscess formation in this study was related to extremely high stability of the emulsion. It is also possible that antigenic differences could explain the differences in systemic reactogenicity between this study and the one evaluating malaria antigens with IFA. In this study the only severe systemic reactions occurred after the second dose in the 4 mg recipients. The rapid onset of headache, chills, nausea, and myalgia within hours after vaccination in this study suggests the vaccine formulation activated innate responses, but the higher severity of reactions after the second vaccination suggests that tissue-resident antigen-specific effectors from the first vaccination may have also contributed to the exaggerated response. The reason why subjects with the most severe systemic reactions did not develop severe local reactions is not known. It is possible this was a coincidence because of the small trial size or may reflect something about the nature of the underlying inflammation responsible for the local and systemic reactions.

Relative to the prior experience with peptide-based vaccines, this product had good immunogenicity. Antibody responses were detectable in 100% of subjects after two immunizations, which is notable for subunit vaccines in general. T cell responses were detected in the majority of subjects despite the early termination and incomplete vaccination schedule and the fact that this study was performed and analyzed during the historical period when both cryopreservation techniques and assay methodology were in transition. Therefore, the frequency and magnitude of the ELISpot and ICS responses in this report may be lower than they would have been if processed and tested with current methodologies. This study was specifically designed to detect HLA-B7-restricted CD8 T-cell responses to the V3 peptide epitope (RPNNNTRKSI). The absence of a ^51^Cr CTL response to this epitope in 4/6 HLA-B7+ subjects tested may be due to the relatively low sensitivity of the assay. In contrast, 5/7 HLA-B7+ subjects had vaccine-induced CD8 T-cell responses by ICS. The two nonresponders may have not had sufficient vaccine-induced immune activation, the assays may have been insensitive, or there may have been other influences related to epitope liberation, processing, or degradation, competing responses to other epitopes, or subject-specific response hierarchies that explain the lack of detectable T cell responses. Three of the 9 HLA-B7-negative subjects also had detectable CD8 T-cell responses. Two of these subjects were HLA-A2+ and could have recognized a variant of the RGPGRAFVTI epitope described in V3 [24], although the exact sequence is not represented in this vaccine. The other subject was HLA-A1, B8, and B57 and the epitope in the vaccine that is restricted by these alleles is not known. These data suggest that peptides can elicit CD8 CTL when formulated with a potent adjuvant. However, it should be kept in mind that control of HIV may require broad responses to multiple epitopes, and eliciting potent epitope-specific CD8 T-cell responses requires more than MHC compatibility. Therefore, restricting the antigenic content of vaccine antigens may limit the host options for selecting and presenting effective T cell targets and will limit the breadth of the response.

This study, AIDS Vaccine Evaluation Group (AVEG) Protocol 020, was designed to enroll 28 subjects to evaluate two doses of the peptide-based vaccine in IFA, but was terminated early because of sterile abscess formation in 4 of the first 24 enrollees. While the peptide-based vaccine was immunogenic, the local reactogenicity was unacceptable for continued development. We show that achieving an immunogenic peptide formulation is feasible in humans, but it may be associated with a significant reactogenicity cost, which in this study was unacceptable. Considering the outcome of this study and the malaria vaccine study, future studies using Montanide ISA-51 as an adjuvant in healthy subjects should be carefully considered from the standpoint of both local and systemic reactogenicity. Overly stable emulsions that may mitigate systemic reactogenicity have the potential to cause unacceptable local reactogenicity including chronic sterile abscesses.

## Supporting Information

Table S1 Sequence of C4-V3 peptides in vaccine.
(0.03 MB DOC)

Table S2 Summary of subjects with sterile abscesses.
(0.04 MB DOC)

Diagram S1 Consort diagram.
(0.05 MB DOC)

Protocol S1 Trial Protocol.
(0.23 MB PDF)

Checklist S1 CONSORT Checklist.
(0.04 MB PDF)
