# Supplementary material for: Immunization with Cocktail of HIV-Derived Peptides in Montanide ISA-51 Is Immunogenic, but Causes Sterile Abscesses and Unacceptable Reactogenicity
Source: PLoS One. 2010 Aug 10;5(8):e11995. doi: 10.1371/journal.pone.0011995 (PMC2919382; doi:10.1371/journal.pone.0011995)
Supplement: Table S1 — Sequence of C4-V3 peptides in vaccine. (0.03 MB DOC) [file pone.0011995.s001.doc]

Table S1. Sequence of C4-V3 peptides in vaccine

| Strain | Sequence of T1SP10 (A) Peptide |
| --- | --- |
| MN | KQIINMWQEVGKAMYATRPNYNKRKRIHIGPGRAFYTTK |
| RF | KQIINMWQEVGKAMYATRPNNNTRKSITKGPGRVIYATG |
| EV91 | KQIINMWQEVGKAMYATRPGNNTRKSIPIGPGRAFIATS |
| CANO | KQIINMWQEVGKAMYATRPHNNTRKSIHMGPGKAFYTTG |
