# Supplementary material for: Immunization with Cocktail of HIV-Derived Peptides in Montanide ISA-51 Is Immunogenic, but Causes Sterile Abscesses and Unacceptable Reactogenicity
Source: PLoS One. 2010 Aug 10;5(8):e11995. doi: 10.1371/journal.pone.0011995 (PMC2919382; doi:10.1371/journal.pone.0011995)
Supplement: Table S2 — Summary of subjects with sterile abscesses. (0.04 MB DOC) [file pone.0011995.s002.doc]

Supplementary Table 2. Summary of subjects with sterile abscesses.

| Subject | Group | # of vaccinations | Days after last vaccination | Clinical Course |
| --- | --- | --- | --- | --- |
| 1 | 4 mg | 2 | 61 | Pain, tenderness, swelling in left shoulder initially thought to be subacromial bursitis from recent floor scrubbing |
|  |  |  | 71 | fluid draining from arm |
|  |  |  | 85 | 6 × 10 cm area of induration over left arm with central 4 cm erythematous nodule; healing eschars; nontender 6×4 cm nodule over right deltoid |
|  |  |  | 150 | 25 ml exudates aspirated from right arm lesion; incision and drainage |
|  |  |  | 157 | incision and drainage left |
|  |  |  | 177 | Resolution |
|  | | | | |
| 2 | 1 mg | 2 | 51 | Throbbing pain and swelling bilaterally |
|  |  |  | 58 | Pain increasing despite warm compresses; Right deltoid 9×9 cm induration with 5.5×7cm erythema; Left 8×8 cm induration, 5×4.5 cm erythema; Pain on palpation |
|  |  |  | 63 | Ultrasound shows 4.7×1.8×4cm fluid on left. 6.1×3×4 cm on right |
|  |  |  | 83 | spontaneous drainage on left. |
|  |  |  | 84 | spontaneous drainage on right |
|  | | | | |
| 3 | 1 mg | 1 | 11 | Mild bilateral soreness beginning |
|  |  |  | 15 | Unable to sleep on left side; 6×2.5 cm induration |
|  | | | | |
| 4 | 4 mg | 1 | 6 | Bilateral deltoid soreness; Reported diffuse muscle soreness from skiing |
|  |  |  | 21 | Continued pain; wakes subject from sleep |
