# Supplementary material for: Immunization with Cocktail of HIV-Derived Peptides in Montanide ISA-51 Is Immunogenic, but Causes Sterile Abscesses and Unacceptable Reactogenicity
Source: PLoS One. 2010 Aug 10;5(8):e11995. doi: 10.1371/journal.pone.0011995 (PMC2919382; doi:10.1371/journal.pone.0011995)
Supplement: Diagram S1 — Consort diagram. (0.05 MB DOC) [file pone.0011995.s003.doc]

**AVEG 020 CONSORT Diagram**

Allocation

Analysis

Follow-Up

Enrolled (n = 24)

Analyzed (n = 21)

Excluded from analysis (n = 0)

Lost to follow-up (n = 1)

Completed scheduled follow-up

(n = 20)

HIV Peptides/IFA (n = 21)

Received 4 planned injections (n = 0)

Received 2 injections (n = 16)

Received only 1 injection (n = 5)

Lost to follow-up (n = 1)

Completed scheduled follow-up

(n = 2)

IFA Control (n = 3)

Received 4 planned injections (n = 0)

Received 2 injections (n = 2)

Received only 1 injection (n = 1)

Analyzed (n = 3)

Excluded from analysis (n = 0)

Randomization
