# Supplementary material for: Immunization with Cocktail of HIV-Derived Peptides in Montanide ISA-51 Is Immunogenic, but Causes Sterile Abscesses and Unacceptable Reactogenicity
Source: PLoS One. 2010 Aug 10;5(8):e11995. doi: 10.1371/journal.pone.0011995 (PMC2919382; doi:10.1371/journal.pone.0011995)
Supplement: Protocol S1 — Trial Protocol. (0.23 MB PDF) [file pone.0011995.s004.pdf]

VERSION 3
DECEMBER 14, 1998

AIDS VACCINE EVALUATION GROUP

PROTOCOL 020

A PHASE I SAFETY AND IMMUNOGENICITY TRIAL OF HIV-1 gp120 C4-V3
HYBRID POLYVALENT PEPTIDE IMMUNOGEN
MIXED IN MINERAL OIL CONTAINING MANNOSE MONO-OLEATE (IFA)

Vaccine Provided by
Wyeth-Lederle Vaccines and Pediatrics

Clinical Trial Sponsored by the
NIH, NIAID, DAIDS, Vaccine and Prevention Research Program
Clinical Development Branch
Bethesda, MD

BB IND 7289*[held by DAIDS]*

<u>Clinical Centers</u>
Vanderbilt University
University of Rochester
University of Washington

<u>Data Coordinating and Analysis Center</u>
The EMMES Corporation

<u>Central Immunology Laboratory</u>
Duke University

Version 1: 07/31/97
Version 2: 08/08/97
Version 3: 12/14/98

CONTACT LIST FOR PARTICIPANTS

Vanderbilt University
Dept. of Medicine [A-4103]
Medical Center North
1162 21st Avenue, South
Nashville, TN 37232

Barney Graham, M.D., Ph.D.*	615-343-3717
Peter Wright, M.D.	615-322-2477
Paul Spearman, M.D.	615-343-5618

University of Rochester
Infectious Diseases, Box 689
601 Elmwood Avenue
Rochester, NY 14642

Michael Keefer, M.D.	716-275-8058
Thomas Evans, M.D.	716-275-5871

University of Washington
AIDS Vaccine Evaluation Unit
Fred Hutchinson Cancer Research Center
1100 Fairview Ave., N., D3-100
Seattle, WA 98109

Lawrence Corey, M.D.	206-667-6702
Julie McElrath, M.D., Ph.D.	206-667-6704

Central Immunology Laboratory
Duke University Medical Center
SORF Building, Room 201
La Salle Street Extension
Durham, NC 27710

Dani Bolognesi, Ph.D.	919-684-3103
Kent Weinhold, Ph.D.	
Thomas Matthews, Ph.D.	
David Montefiori, Ph.D.	

NATIONAL INSTITUTE OF ALLERGY AND INFECTIOUS DISEASES
Division of AIDS
Vaccine and Prevention Research Program
Clinical Development Branch

Solar Building
6003 Executive Boulevard, MSC 7620
Bethesda, MD 20892-7620
FedEx Zip Code 20852

Dale Lawrence, M.D., M. P. H.**	301-435-3755
Barbara Savarese, R.N.**	301-435-3750
Mary Allen, R.N., M.S.	301-402-2310
Suzanne Gallo	301-435-3748

Pharmacy and Regulatory Affairs Branch

Scharla Estep, M.S., R.Ph.	301-435-3746

DATA COORDINATING AND ANALYSIS CENTER
The EMMES Corporation
11325 Seven Locks Road, Suite 214
Potomac, MD 20854

Donald Stablein, Ph.D.+	301-299-8655
Mark Wolff, Ph.D.	
Carol Smith, M.P.H.++	

VACCINE MANUFACTURER
Wyeth-Lederle Vaccines and Pediatrics

- One Great Valley Pkwy
  Malvern, PA 19355

  Richard Ginsberg, M.D., M.S. 610-647-9452

- 211 Bailey Road
  West Henrietta, NY 14586

  John Eldridge, Ph.D 716-273-7661

Duke University
Duke University Medical Center
Department of Medicine, Rm. 1102
Duke Hospital North - Erwin Road
Durham, NC 27710

Barton Haynes, M.D.	919-684-5384

* Study Chair
** Medical Officer
\+ Study Statistician
\++ Study Coordinator

TABLE OF CONTENTS

		Page
	Protocol Summary	iii
1.0	Purpose of Study	1
2.0	Introduction and Rationale	2
3.0	Background on Vaccine Preparations	3
4.0	Experimental Design	5
5.0	Data and Safety Monitoring	8
6.0	Statistical Analysis	10
7.0	Randomization and Blinding	12
8.0	Selection of Subjects	13
9.0	Immunization Procedures	15
10.0	Studies to be Performed	18
11.0	References	23
12.0	Informed Consent Procedures with Sample Consent Forms	24
13.0	Modifications to Protocol	35
APPENDICES		
A	Definition of Risk Categories	45
B	Screening Procedures for HIV Vaccine Trial Volunteers	47
C	Algorithm for Determination of HIV Infection	51
D	EmulsiFlex 1000™ Operation and Cleaning Procedures	52
E	Time Course for Immunogenicity Determinations - REVISED	58
F	Calendar of Studies - REVISED	59

PROTOCOL SUMMARY

Protocol 020: Phase I Safety and Immunogenicity Trial of HIV-1 gp120 C4-V3 Hybrid Polyvalent Peptide Immunogen Mixed in Mineral Oil Containing Mannose Mono-oleate (IFA)

Subjects: Healthy, HIV-uninfected adult volunteers with lower risk for HIV-1 infection, 50% must have the HLA-B7 phenotype. Total of 28 volunteers.

Schema:

Immunizations scheduled at Months 0, 1, 6 and 12		
Group	C4-V3 Peptides in IFA	IFA Alone
I	* 12 (1 mg total, 250 μg of each peptide)	
II	* 12 (4 mg total, 1 mg of each peptide)	
III		** 4
TOTAL	24	4

* 6 subjects will be HLA-B7 phenotype ** 2 subjects will be HLA-B7 phenotype

Estimated Time Period: 18 months

Monitoring of Trial: AVEG Data and Safety Monitoring Board

Sponsoring Agency: Clinical Development Branch, Vaccine and Prevention Research Program (VPRP), Division of AIDS (DAIDS), National Institute of Allergy and Infectious Diseases (NIAID), National Institutes of Health (NIH)

Clinical Sites: Vanderbilt University, University of Rochester, and University of Washington

Data Coordination: The EMMES Corporation

Central Immunology Laboratory: Duke University

1.0 PURPOSE OF STUDY

The purpose of the study is to evaluate the safety and immunogenicity of a polyvalent HIV-1 C4-V3 synthetic peptide mixture formulated in Incomplete Freund's Adjuvant (IFA, mineral oil containing mannose mono-oleate). Four synthetic peptides based on the HIV-1 clade B strains MN, EV91, RF, and CANO are included in the candidate vaccine. Each of these four component peptides consists of two sections or parts. The first, the "C4" section, is a 15 amino acid sequence corresponding to a potent activator of anti-HIV memory helper T cells present in the fourth conserved region of HIV-1 gp120. The second part, the "V3" section, is a 24 amino acid sequence which corresponds to the V3 loop region of gp120 of the particular HIV-1 strain. The V3 region is an established B cell epitope recognized by anti-HIV neutralizing antibodies, as well as being an HLA-B7 restricted CTL epitope. The same “C4" sequence 15 is used in all four of the pooled fusion peptides, while the V3 portion of the fusion peptide is unique for each strain. The candidate vaccine, hereafter referred to as C4-V3 peptides, is to be given by intramuscular injection to healthy, HIV-1 uninfected adult volunteers at low risk of infection with HIV-1.

1.1 Primary Objective

To evaluate the safety of the C4-V3 peptides formulated in IFA in HIV-1 uninfected volunteers.

1.2 Secondary Objectives

To evaluate the humoral and cellular immune responses to the C4-V3 peptides by determining whether immunization induces one or more of the following:

A. Neutralizing antibodies to HIV-1 MN and RF

B. Cross-neutralizing antibodies to primary isolates of HIV-1

C. HIV-1 antigen-specific lymphoproliferation

D. CD8+ and CD4+ cytotoxic T lymphocyte (CTL) activity specific for HIV-1 gp120 or V3 peptides corresponding to the vaccine strains of HIV-1

E. Induction of HLA-B7 and HLA-A2 restricted CD8+ CTLs

f. Induction of HIV-specific DTH responses

2.0 INTRODUCTION AND RATIONALE

AIDS has emerged as a world-wide, uncontrolled public health problem and is associated with extensive morbidity and mortality throughout the world. Although recent advances have been made in antiviral therapy against AIDS, there is currently no cure for AIDS. It is likely that ultimate control of the disease will depend on the development of safe and effective vaccines against HIV.

HIV-1 sequence diversity in the principle neutralizing domain of gp120 (the V3 gp120 envelope loop region) and rapid V3 loop sequence mutation rate is a major obstacle to overcome for vaccine development (1-3). Nonetheless, studies continue to show the critical role that gp120 V3 region plays in generating anti-HIV neutralizing antibodies (4). Moreover, it has recently been shown that approximately 50% of current HIV isolates share a consensus of V3 sequences that is similar to the HIV MN isolate, and that approximately 80% of HIV isolates in the US share one of the 4 most common HIV sequences (1-3). Moreover, two of these sequences, GPGRAF and IHIGPGRA, have induced widely cross-reactive HIV neutralization antibodies in animals (5,6). An HLA-B7 restricted CTL epitope has also been defined in the V3 region (17).

The T1-SP10MN(A) peptides are potent immunogens in primates, while addition of the gp41 fusogenic (F) domain to the peptides yielded a potent tolerogen in primates. These data suggest that T1-SP10(A) peptides may be immunogenic in humans and induce anti-HIV-1 neutralizing antibody responses. Moreover, the T1-SP10MN(A) may induce CTL responses. Since the MHC Class I-restricted CTL epitope in the V3 loop has been reported to be restricted to HLA-B7, the ability of T1-SP10MN(A) and related peptides to induce MHC Class I CTL can be tested in man. A similar product prepared with the gag epitope of SIV has been shown to induce CD8+ Class I-restricted CTL responses in rhesus monkeys (18).

To address these issues and to design an immunogen capable of inducing a broad range of cross-reactive neutralizing antibodies in man, 4 sequences of the HIV-1 V3 gp120 loop have been chosen from divergent HIV-1 isolates from the US, and together represent ≈80% of North American HIV-1 strains (1-3). It is now clear that any HIV-1 vaccine based on V3 loop antibodies will require multiple V3 loop sequences to be included. Moreover, the data in rhesus monkeys and chimpanzees showing that different outbred primates vary in their ability to recognize neutralizing epitopes of the V3 loop, suggesting that not all individuals of an outbred cohort of humans will respond to each V3 peptide at the same site.

A Phase I safety and immunogenicity study, DATRI Protocol 010, of the C4-V3 peptide candidate AIDS vaccine has been completed in 10 HIV-infected, HLA B7-positive volunteers who had CD4 cell counts >500/mm^3 at time of enrollment in the study. Eight volunteers received intramuscular injections of the immunogen emulsified in Incomplete Freund's Adjuvant (IFA) and the other two received the IFA placebo alone, given at 0, 4, 8, 12 and 24 weeks. Each C4-V3/IFA injection contained a total peptide content of 2 mg, 0.5 mg of each of the 4 fusion peptides. In 4/8 C4-V3/IFA recipients, a four-fold increase was observed in ELISA titers to at least 3 of the 4 C4-V3 peptides based on the four vaccine HIV strains. Similarly, 4 of 8 C4-V3/IFA recipients had a four-fold rise in neutralizing antibodies to either HIV—MN, HIV—RF or HIV—4489.5 laboratory-adapted HIV isolates. One of 2 controls had a rise in titer to only 1 immunizing peptide (the other control subject had rises to 0 of 4 immunizing peptides), and 0 of 2 controls had rises in neutralizing antibody levels. Lymphoproliferation stimulation indices increased ≥5 fold at least one of the immunizing peptides in 5 of 5 vaccine recipients and 0 of 2 IFA control patients. Adverse events consisted of grade I injection site reactions in 6 volunteers—4 vaccinees and 2 placebo recipients.

3.0 BACKGROUND ON VACCINE PREPARATIONS

3.1 Vaccine

In previous studies, it has been shown that construction of an HIV env C4-V3 synthetic peptide T1-SP10(A) containing aa303-327 of HIV gp120 V3 loop [SP10(A)] and aa428-443 of HIV gp120 (T1) serves as a potent T cell immunogen for induction of activation of anti-HIV memory T helper cells and B cell immunogen for anti-HIV neutralizing antibodies *in vivo* (7-10). The T1-SP10(A) peptide induces anti-HIV neutralizing antibodies in mice, goats and rhesus monkeys (7-11,13), induces anti-HIV MHC Class I-restricted CTL in mice (10) and monkeys (18), and induces anti-HIV T helper cell responses in mice, goats, rhesus monkeys, and chimpanzees (7-13). The CTL epitope in the V3 loop region is known to be restricted in man by HLA-B7 (17).

The adjuvant to be used in this clinical trial contains 10% mannose mono-oleate in mineral oil and is commonly referred to as incomplete Freund's adjuvant (IFA). Extensive experience with IFA has been accumulated in hundreds of thousands of human injections without significant toxicities. An IFA preparation identical to the one to be used in this study has been employed in HIV-infected volunteers in a small trial of soluble CD4 (Dr. Norman Levin, personal communication) and is being used in therapeutic trials of an envelope-depleted whole killed HIV-1 candidate vaccine (Immune Response Corporation).

The HIV-env C4-V3 peptides are dissolved in pH 5.5 distilled water + 5% dextrose and then emulsified in the IFA for use. This formulation has been tested in rabbits, rhesus monkeys, and in a small Phase I trial in HIV-1 infected volunteers (NIAID DATRI Protocol 010). The standard rabbit toxicity study employed 32 male and female New Zealand White rabbits. The peptides were given with and without IFA and each rabbit received 2 IM injections, one in each thigh, on days 0, 21, 42, 63, and 84. Injections were at the same (marked) site for the first 4 immunizations and then given at a site 2 cm anterior to the other injections at day 84. Throughout the study, the rabbits were healthy, gained weight, had only minor local reactions to the injections, and had normal serum chemistries and hematology results. At necropsy, no significant differences or abnormalities were seen in the different treatment groups with respect to gross organ or injection site biopsy histopathology. All rabbits receiving the C4-V3 peptides made an antibody response and the response was higher in those animals who received the peptides emulsified in IFA.

The study in two rhesus monkeys used three injections containing 8 mg of the polyvalent HIV-1 C4-V3 vaccine. Both monkeys had local erythema and swelling after the second injection. One had lethargy and decreased use of the right leg following the third injection. At necropsy, granulomatous inflammation was noted at the injection site and to a lesser extent in the regional lymph nodes.

In earlier preclinical toxicology studies, it was found that the technique used to prepare the vaccine did not always result in a stable emulsion. In animals injected with the peptide product in an unstable emulsifier, granulomatous inflammation with intracellular vacuoles (lipid) were found in both lung and liver. This was not seen in the toxicology studies described above using vaccine prepared in a manner to achieve stable emulsification, but the earlier finding led to the decision to use the EmulsiFlex 1000™ apparatus for vaccine preparation, testing the emulsification stability prior to use, and exclusion of subjects with prior lung disease or excessive alcohol consumption.

In a Phase I double-blinded pilot study conducted by the National Institute of Allergy and Infectious Diseases, 4 of 8 C4-V3/IFA recipients and 2 IFA placebo recipients had grade I injection site reactions; no other local or systemic reactions other than mild or moderate were observed over the course of 5 immunizations.

3.2 Placebo

The placebo preparation will be mineral oil containing 10% mannose mono-oleate (IFA) diluted 1:1 with sterile PBS.

4.0 EXPERIMENTAL DESIGN

The study is a multicenter, randomized, controlled, double-blinded Phase I trial conducted at three clinical sites to evaluate the safety and immunogenicity of a mixture of C4-V3 peptides in IFA adjuvant. Twenty-eight HIV-1 uninfected healthy adult volunteers will be randomly allocated to receive 1.0 ml total dose of immunogen or IFA adjuvant alone, divided into two 0.5 ml deltoid muscle (IM) injections, and administered as shown in the Protocol Summary (page iii). Twenty-four volunteers will receive the test vaccine (12 will receive 250 μg of each peptide in a mixture and 12 will receive 1 mg of each peptide in a mixture), and 4 will receive placebo. Six vaccine recipients at each dose level and 2 placebo recipients will have the HLA-B7 phenotype. Additional volunteers may be recruited to compensate for any who are lost to follow-up.

Each volunteer will undergo informed consent and prescreening evaluation. If eligibility criteria are met, each volunteer will receive intramuscular injections of the vaccine or placebo at 0, 1, 6, and 12 months. All immunizations will be administered by the intramuscular route at 2 sites (right and left deltoids). The immunogen used in this study will be a combination of C4-V3 peptides including sequences from MN, EV91, RF, and CAN0 HIV strains. The peptide mixture will include either 250 μg or 1 mg of each component peptide for a total dose of either 1 or 4 mg. It will be administered in Incomplete Freund's Adjuvant (IFA) mixed with the immunogen in a 1:1 volume/volume mixture. The total volume for each immunization will be 1.0 ml.

Volunteers will be evaluated according to the schedule listed in Section 10, *Studies to be Performed*. All concomitant medications must be recorded during this trial. Immunosuppressive agents should not be administered during the study unless medically necessary.

The two major immunogenicity endpoints will be: a) the generation of anti-HIV-1 neutralizing antibodies (NT) against HIV MN and RF (or HIV isolates with similar V3 regions to these isolates) and b) generation of HLA-B7 restricted anti-HIV CTL. The induction of V3-specific CTL activity restricted by the HLA-A2 phenotype will also be measured. (19).

4.1 Safety Evaluation *[see Section 5]*

Safety will be evaluated by monitoring of volunteers for local and systemic adverse reactions during the course of the trial. Volunteers will be closely monitored for the first two weeks after each immunization, and followed for a total of 18 months after the initial immunization. In addition to assessment of local and systemic reactions, hematologic and chemistry parameters of liver and kidney function will be monitored.

4.2 Immunogenicity Determinations *[Refer to Sections 10.2 and 10.3 and Appendices E and F for details of assays to be performed]*

Antibody responses will be evaluated by HIV-specific ELISA, neutralization and fusion inhibition assays, peptide antibody binding assays to V3 loop peptides and blocking of gp120 binding to CD4. Cellular immune assays including lymphoproliferative assays and Class I restricted CTL activity assessments will be performed on all volunteers.

Peripheral blood mononuclear cells (PBMC) will be separated and cryopreserved at the times indicated in the time course chart. Note that the procedures for cryopreservation and thawing are detailed in Section 5 of the MOP. In addition, an aliquot of PBMC isolated pre-vaccination will be EBV transformed using the specified procedures [see Section 5 of the MOP]. Lymphocyte proliferation assays will be performed using cryopreserved PBMCs obtained on the study days

specified in the protocol and on the time course chart; details of the procedure are also provided in Section 5 of the MOP. Additional measures of cellular immunity including SCID-Hu mouse studies may be conducted at certain AVEUs or by subcontract as ancillary studies.

4.3. <u>Routine Tests</u> *[Refer to Section 10.3 and Appendix F for the schedule of tests to be conducted]*

Routine tests to be conducted for all volunteers include the following:

1. Routine serum chemistries (renal and hepatic function) [AVEUs]
2. Complete blood count [AVEUs]
3. Serum or urine HCG for women [AVEUs]
4. FDA-approved HIV ELISA [AVEUs]
5. Routine urinalysis [AVEUs]
6. HLA typing, Class I only [AVEUs]
7. Initiation of an EBV transformed B cell line [AVEUs]

4.4 <u>Detection of Intercurrent HIV Infection</u>

This is a vaccine containing an envelope protein of the HIV-l virus. Therefore, vaccine-induced immune responses in study volunteers detected by screening serologic assays could potentially be confused with natural infection. Several precautionary measures will be taken to distinguish between responses:

1) Only volunteers at lower risk for HIV-infection (AVEG risk groups A and B as defined in Appendix A) will be considered for enrollment. Volunteers will be counseled frequently during the trial on avoidance of HIV infection.

2) Volunteers will have frequent clinical evaluations for signs or symptoms of an acute HIV infection syndrome [see Section 10.3]. An intercurrent illness consistent with HIV-1 infection or any history of higher risk behavior would prompt a diagnostic work-up including HIV-1 serology and HIV-1 DNA PCR (± HIV culture). Refer to Appendix C.

3) Periodic HIV ELISAs will be performed [see Section 10.3]. If intercurrent HIV infection is suspected, further diagnostic work-up will be performed. [See Appendix C] Periodic T cell subset analyses will also be performed; any wide fluctuations outside the range of normal variation which could suggest intercurrent infection will prompt further diagnostic work-up for HIV-1.

4) Long-term follow-up will be important to rule out subsequent HIV infection or concerns in volunteers whose HIV assays are positive by immunoassay at the end of the study. All volunteers who have positive HIV-1 serology at end of study, as measured by the Abbott HIV 1,2 kit, will be offered follow-up HIV-1 diagnostic testing (HIV ELISA, Western Blot, PCR ± culture) periodically as medically/socially indicated (approximately every six months). This follow-up will continue until the serologic studies no longer yield positive results.

4.5 Additional Phases

Depending on the safety and immunogenicity results, volunteers may be offered an additional boost of the same or a different immunogen at a later time point. Volunteers may elect to discontinue their participation at any time. An additional informed consent will be required for any additional injections given beyond the planned immunizations.

Long-term follow-up of volunteers to determine the duration of immune responses and possible social risks and other safety parameters will be undertaken. At the present time, it is anticipated that annual contact will be maintained for at least 5 years.

5.0 DATA AND SAFETY MONITORING

5.1 Risks

The AIDS Vaccine Data and Safety Monitoring Board (AVDSMB) will monitor the progress of the trial. The Board will periodically review data from the trial, with particular emphasis on monitoring the following adverse reactions.

a. Local toxicity at the site of injection: e.g. pain, tenderness, erythema, induration, regional lymphadenopathy

b. Systemic toxicity: e.g., anaphylaxis, fever, myalgia, malaise, headache, immune complex or hypersensitivity reactions

c. Hematologic: CBC with differential, platelets

d. Hepatic and Renal: ALT, creatinine, GGT (gamma glutamyl transpeptidase), urinalysis

e. Immunologic: CD4 and CD8 lymphocyte number and percentage, lymphocyte proliferation responses to HIV antigens and recall antigens

f. Other reactions: dermatologic, neurologic, gastrointestinal (nausea/vomiting, diarrhea)

Female participants will be cautioned of the unknown hazard of study products to a fetus and advised to use adequate birth control methods for the duration of the study.

5.2 Adverse Experience Reporting Requirements

Adverse experiences that meet Serious Adverse Experience (SAE) Reporting Requirements set forth in the Division of AIDS SAE Reporting Manual for Prevention Trials Programs must be reported on the SAE Report Form and submitted immediately by the sites to the Regulatory Operations Center (ROC) Adverse Experience Report (AER) Office. Reporting criteria and time frames are contained in the SAE Reporting Manual. Completed SAE Forms should be submitted as follows:

1. ROC AER Office Tel: 800-537-9979
Fax: 800-275-7619

and

2. AVEG Data Coordinating and Analysis Center (DCAC)
Tel: 301-299-8655
Fax: 301-299-3991

The ROC AER Office will forward SAE Forms to NIAID Medical Officers and inform the PRAB Safety Specialist at DAIDS Pharmacy and Regulatory Affairs Branch (PRAB). Clinical sites may be contacted by the ROC for additional information needed for IND Safety Reports.

The DCAC will expedite the necessary information flow between the AVEUS, DAIDS, the product manufacturer and the AVDSMB. If a technical problem arises with the 800 number, the NIAID Medical Officer can be reached at (301) 496-8200.

NOTE: All adverse experiences, regardless of severity, must also be reported on the appropriate Case Report Forms (CRFs) and submitted to the DCAC according to standard operating procedures.

5.3 Stopping Rules

Grade 2 or 3 events (as defined in the *ROC SAE Manual)* assessed possibly, probably or definitely related to study product: If **two** such Grade 2 or 3 similar events are reported, further immunizations will be temporarily held for all participants. A Protocol Team conference call will be convened, and a decision regarding continued enrollment and/or immunizations will be made.

Grade 4 events (as defined in the *ROC SAE Manual)* assessed possibly, probably or definitely related to study product: If **one** such Grade 4 event is reported, further immunizations will be temporarily held for all participants. A Protocol Team conference call will be convened, and a decision regarding continued enrollment and/or immunizations will be made.

6.0 **STATISTICAL ANALYSIS**

This study is a Phase I randomized trial to evaluate the safety and immunogenicity of HIV-1 gp120 C4-V3 hybrid polyvalent peptide immunogen, mixed in mineral oil containing mannose mono-oleate (IFA). Twenty-eight volunteers will be enrolled in the study, 12 of whom will receive 4 mg of peptide mixture in Incomplete Freund's Adjuvant (IFA), 12 of whom will receive 1 mg of peptide mixture in IFA, and 4 will receive IFA alone.

SAFETY

Endpoints for safety evaluations of vaccine can be grouped into two broad classes. The first are life threatening reactions to vaccine. Such reactions cannot occur with measurable frequency in any vaccine which can be considered for use. The second group of safety endpoints are severe reactions to vaccine, which are not life threatening but may be temporarily incapacitating (for example, losing a day of work), and therefore could make a vaccine impractical for large scale use if they occur in more than a small proportion of cases. Because both vaccine and placebo groups will receive Incomplete Freund's Adjuvant, the estimates of the overall frequency of life-threatening or severe reactions are important, as well as comparisons between the vaccine and placebo groups.

For either of these types of reactions to vaccine or placebo, the table below gives the 95% upper confidence bounds for their incidence in the population as a function of the number of these reactions observed.

95% upper confidence bounds for the incidence of severe reactions
by number of reactions out of 24

Number of reactions	Upper confidence bound
0	0.12
1	0.18
2	0.24
3	0.29
4	0.34
5	0.39

For severe reactions, assuming a reference rate of 0.05 in the placebo group, and using a two-sided test of proportions with type I error 5%, a comparison of the rates of severe reactions between vaccine and placebo groups has 80% power against an increase to 0.62 in the vaccine group. Hence this study will be able to detect reliably only large differences in the rate of severe reactions between the vaccine and placebo groups.

IMMUNOGENICITY

The analyses of vaccine immunogenicity will be based on measures of both humoral and cellular immune responses. The assays of humoral immune response include ELISA assays to individual vaccine-specific antigens. Functional assays include multidose neutralization assays against MN and RF, and field HIV isolates, fusion inhibition assays, and blocking of gp120 binding to CD4. Measurement of peptide-specific CTL activity will also be made.

<u>Comparisons of neutralization titers.</u> The comparison of neutralization titers will be used to demonstrate the power of this study for detecting differences in vaccine immunogenicity. Data from current AVEG protocols shows that $\log_{10}$ neutralization titers are approximately normally distributed with standard deviation approximately equal to 0.434. A comparison of vaccinated (n=24) versus placebo (n=4) volunteers with respect to this endpoint can detect a difference of 0.7 in $\log_{10}$ neutralization titer using a two-sided t-test with size 5% and 80% power. This corresponds to a slightly more than 5-fold higher neutralization titer in vaccinated volunteers compared to placebo volunteers. For the comparison between 1 mg and 4 mg vaccine dose groups, a difference of 0.51 $\log_{10}$ neutralization data can be detected.

<u>Comparisons of Cytotoxic T-lymphocyte (CTL) responses</u>. Assuming a reference rate of 0.05 in the placebo group, and using a two-sided test of proportions with type I error 5%, a comparison of the rates of HIV-specific CTL activity between vaccine and placebo groups has 80% power against an increase to 0.62 in the vaccine group. For the comparison between 1 mg and 4 mg vaccine dose groups, using a 50% positive rate as reference, a difference of 0.46 can be detected with type I error 5% and 80% power.

7.0 RANDOMIZATION AND BLINDING

The randomization sequence will be obtained by computer-generated random numbers and provided to each AVEU by the DCAC using the procedures described in the AVEG *Manual of Operations*. The randomization code is kept at each institution by the pharmacist with primary responsibility for drug dispensing. Vaccine will be supplied by the vaccine manufacturers and made available to the AVEG by the Clinical Research Product Management Center (CRPMC). Test preparations will be provided to the investigators and clinic staff in a blinded fashion by the study center pharmacist. Unblinding will occur at the end of the study following AVEG policy, as defined in the AVEG *Manual of Operations*.

Randomization will be stratified so that 50% of the volunteers in the treatment and placebo groups will have the HLA-B7 phenotype.

8.0 SELECTION OF SUBJECTS

Subjects will be healthy HIV-1 uninfected (seronegative) adults who fully comprehend the purpose and details of the study. Subjects who themselves or whose sexual partners have identifiable higher risk behavior for HIV-1 infection will not be eligible for these studies. Higher risk behavior will be determined by a prescreen series of questions designed to identify risk factors for HIV-1 infection (Appendix A). Only volunteers meeting the criteria for Risk Groups A or B will be enrolled in this trial. A face sheet (Volunteer Profile Form, Appendix B) will be used to obtain identifying and baseline demographic data about each volunteer. After completion, the face sheet will be removed and filed separately and all additional forms will be identified by volunteer number only. An assessment of absolute exclusion criteria using self-administered and interview questions will follow (Appendix B). After volunteers sign the consent form for pretrial eligibility tests, investigators will proceed with phlebotomy, medical history, physical examination, and final questions regarding sexual behavior and other practices. Eligibility determination for the trial will be dependent on results of laboratory tests and answers to these self-administered and interview questions.

8.1 Inclusion Criteria

- Age: 18-60 *[No more than 10% of the volunteers to be over age 50]*
- Sex: Male or Female *[For females, negative pregnancy test at time of entry and assurance that adequate birth control measures will be used for one month prior to immunization and for the duration of the study.*]
- Normal history and physical examination
- Lower risk sexual behavior as defined by AVEG
- Normal complete blood count and differential defined as:
  - Hematocrit ≥ 34% for women; ≥ 38% for men
  - White count ≥ 3500 cells/mm^3 with normal differential
  - Total lymphocyte count ≥ 800 cells/mm^3
  - Absolute CD4 count ≥ 400 cells/mm^3
  - Platelets (150,000-550,000/mm^3
- Normal ALT (≤ 1.5 x institutional upper normal limit)
  and Creatinine (≤ 1.6 mg/dl)
- Normal urine dipstick with esterase and nitrite
- Negative for Hepatitis B surface antigen
- Negative ELISA for HIV within 8 weeks of immunization
- Availability for follow-up for planned duration of the study (18 months)

SPECIAL INCLUSION CRITERIA FOR THIS STUDY

- Normal GGT (≤ 1.5 x institutional upper normal limit)
- Normal chest x-ray within 4 weeks prior to initial immunization
- Agree to limit alcohol intake during study participation to less than the equivalent of 1 oz of 100 proof per day (4 oz. glass of wine or 12 oz. of beer per day)

  NOTE: In early toxicology studies performed in rabbits and monkeys in which the vaccine was not properly emulsified, granulomatous inflammation was noted in both lung and liver. Although the vaccine prepared for the study is being tested for stability prior to administration, this criterion is being employed to avoid difficulties in interpretation of safety data.
- A minimum of 50% of the volunteers will have the HLA-B7 phenotype

8.2 Exclusion Criteria

- History of immunodeficiency, chronic illness, malignancy, autoimmune disease, or use of immunosuppressive medications. Individuals with a history of cancer are excluded unless there has been surgical excision followed by a sufficient observation period to give a reasonable assurance of cure
- Medical or psychiatric condition or occupational responsibilities which preclude subject compliance with the protocol. Specifically excluded are persons with a history of suicide attempts, recent suicidal ideation, or who have past or present psychosis
- Subjects with identifiable higher risk behavior for HIV infection as determined by screening questions designed to identify risk factors for HIV infection; specific exclusions include:
  - History of injection drug use within the last 12 months prior to enrollment
  - Higher or intermediate risk sexual behavior as defined by the AVEG (i.e., meeting the criteria for AVEG Risk Groups C or D) [see Appendix A]
- Live attenuated vaccines within 60 days of study

  NOTE: Medically indicated subunit or killed vaccines (e.g., influenza, pneumococcal) are not exclusionary, but should be given at least 2 weeks away from HIV immunizations.
- Use of experimental agents within 30 days prior to study
- Receipt of blood products or immunoglobulin in the past 6 months
- Active syphilis

  NOTE: If the serology is documented to be a false positive or due to a remote (>6 months) treated infection, the volunteer is eligible.
- Active tuberculosis

  NOTE: Volunteers with a positive PPD and a normal chest X-ray showing no evidence of TB and not requiring INH therapy are eligible.
- History of anaphylaxis or other serious adverse reactions to vaccines
- History of serious allergic reaction to any substance, requiring hospitalization or emergent medical care (e.g., Steven-Johnson syndrome, bronchospasm, or hypotension)
- Prior receipt of HIV-1 vaccines or placebo recipient in a previous HIV vaccine trial
- Pregnant or lactating women

SPECIAL EXCLUSION CRITERIA FOR THIS STUDY

- History of lung disease

9.0 IMMUNIZATION PROCEDURES

9.1 Vaccine Ordering

Vaccine products for this trial will be available through the NIAID Clinical Research Product Management Center (CRPMC). The AVEG pharmacist can obtain study vaccine for this protocol by ordering it from the CRPMC by following the ordering instructions in the *Pharmacy Guidelines and Instructions for AIDS Clinical Trials Group* in the section on Investigational Drug Control.

9.2 Vaccine Supplies, Preparation Instructions and Administration of Vaccine

9.2.1 Materials and Equipment *[See Appendix D, Section 5]*

- EmulsiFlex 1000™: 2 (two) sterile EmulsiFlex 1000™ units. 1 (one) dedicated to HIV-1 C4-V3 polyvalent peptide/Montanide ISA-51 mixture and 1 (one) dedicated to Montanide ISA-51 /saline mixture (placebo)
- 1 (one) HIV-1 C4-V3 polyvalent peptide vial [0.75 ml (750 μl)] containing 2 mg per 0.5 ml or 8 mg per 0.5 ml
- 1 (one) vial containing sterile 0.9% NaCl for injection
- 1 (one) 3.0 ml ampoule containing Montanide ISA-51 (IFA)
- 1 (one) plastic tuberculin syringe with detachable 25 gauge 5/8Ó needle
- 2 (two) sterile 5 ml Luer-Lock glass syringes [Popper & Sons, Inc. MicroMate]
- 3 (three) sterile 1 ml Luer-Lock glass tuberculin syringes [Popper & Sons, Inc. MicroMate]
- 2 (two) sterile 20g 1-1/2" needles
- 2 (two) sterile 22g 1-1/2" needles

9.2.2 Storage

Single-use vials of immunogen, Montanide ISA 51 and the saline are to be refrigerated at 2-8° C. DO NOT FREEZE. DO NOT SHAKE.

9.2.3 Disposal

Partially-used and empty vials of vaccine should be destroyed on site by crushing or incineration according to Institutional Standard Operating Procedures. All expired or unused vaccine vials must be returned to the CRPMC. Study supplies, including partially-used vials, may not be administered to other subjects or used for *in vitro* or animal experiments.

9.2.4 Dose preparation

See Appendix D, Instructions for Assembly/Disassembly Operation and Cleaning, for the preparation of an emulsion using the Kirkland EmulsiFlex 1000™ and for detailed instructions on the preparation by emulsification of the 1 mg, 4 mg and placebo doses. The procedure results in an emulsion containing either l mg/ml or 4 mg/ml of peptides for the 1 mg and 4 mg doses, respectively, or a 1:1 saline/Montanide ISA-51 mixture for the placebo. Each dose will consist of a total volume of 1.0 ml provided in 2 syringes containing 0.5 ml each of the appropriate emulsion; 0.5 ml will be given in each arm.

9.2.5 Dosing administration

1. Attach the 22g needle, one to each syringe. Hold both barrel and plunger of each glass syringe to avoid sudden separation of the plunger syringe assembly, resulting in loss of contents. Hold each syringe with needle up and carefully expel any air in the syringe.
2. Inject 0.5 ml intramuscularly (IM) deep into each of the left and right deltoid muscles. Therefore, the total volume for each immunization is 1 ml.

9.3 Intradermal Skin Test Protocol *[See Section E for skin test placement]*

Materials required

- Candin™ (*Candida albicans* skin test antigen for cellular hypersensitivity, distributed by ILK Laboratories, 800-325-7354)
- Tetanus toxoid, fluid, USP (Connaught Laboratories)
- Diluent for allergenic extract (0.03% HSA in saline, preserved with phenol), any manufacturer, 1.8 ml vial
- HIV-1 C4 V3 polyvalent peptide skin test antigen and diluent
- Sodium chloride for injection, USP

A. Candida: Preparation and administration

Store according to manufacturer's instructions. Give full strength. Administer 0.1 ml of antigen intradermally. *[See Section E below]*

B. Tetanus toxoid (TT): Preparation and administration

Prepare a 1:10 dilution of TT fluid for use as described below:

1. Remove 0.2 ml of undiluted TT (4 LFU/0.5 ml) from the stock vial and add to 1.8 ml of diluent for allergenic extract, yielding a final volume of 2.0 ml.
2. Mix by gently rocking or swirling the vial.
3. The vial will now contain 20 doses (each 0.1 ml) of a 1:10 dilution of TT
4. Draw up 0.1 ml of the 1:10 dilution of TT in a tuberculin syringe and administer 0.1 ml with a 27 gauge needle, intradermally. *[See Section E below]*
   The diluted TT is stable for 30 days at 2-8° C.

C. HIV-1 C4-V3 polyvalent peptide skin tests: Preparation and administration

HIV-1 C4-V3 polyvalent skin test is supplied in single use vials containing 0.5 ml of solution with a concentration of 100 μg/ml (25 μg of each of the four peptides in 1 ml of water). The skin tests will be given at doses of 1 μg and 10 μg intradermally.

One Microgram (1 μg) Dose

Prepare a 1:10 dilution of the skin test antigen by withdrawing 4.5 ml of the provided diluent (saline) and add to the antigen concentrate vial, yielding a 10 μg/ml solution. MIX WELL. Use within 1 hour. Withdraw 0.1 ml and inject intradermally.

Ten Microgram (10 μg) Dose

The skin test antigen is used at full strength. Withdraw 0.1 ml and inject intradermally.

D. Sterile saline control

Administer 0.1 ml intradermally. *[See Section E below]*

E. Administration of skin test antigens

- The skin test sites for the 5 skin test reagents are shown in Figure 1 below.

RIGHT FOREARM	LEFT FOREARM
Elbow	Elbow
O Candida Skin Test	
O Tetanus Skin Test	O C4-V3 1 μg
O Saline	O C4-V3 10 μg
Wrist	Wrist

NOTE: The site of each skin test antigen injection should be at least 3 cm away from adjacent skin test site.

- Cleanse the skin with 70% alcohol. Allow to dry; do not blow on the skin.
- Using a new ½ inch 27 gauge needle and a tuberculin syringe, withdraw 0.1 ml of the antigen solution. Take care to exclude air bubbles and to ensure that the lumen of the needle is filled.
- With the bevel directed upward, inject **0.1 ml** intradermally (in the same manner as a Mantoux test), forming a small intermediate wheal. Needle withdrawal should be delayed a few seconds to prevent leakage.
- Subjects will be monitored in the clinic for 30 minutes after the skin tests are placed for signs of acute reaction, including a local wheal and flare. Subjects will return to the clinic 48 hours after implantation for assessment of local reaction and adverse events.

F. Measurement of Induration

Perpendicular Diameter (mm)

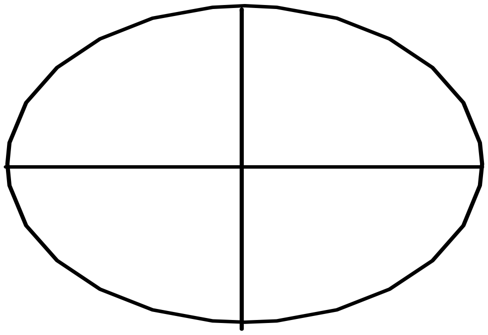

Greatest Diameter (mm)

- To measure induration, it is recommended that the clinician place a ball point pen 1-2 cm outside the area of induration and lightly draw a line toward it, stopping when resistance is met. A short perpendicular line at the point of resistance marks the edge of the induration. The technique is repeated to mark the largest diameters of the induration and the distance between marks is measured in millimeters with a flexible ruler. The mean diameter (mm) will be the mean of the greatest diameter and the perpendicular diameter. **(greatest diameter + perpendicular diameter)/2**
- A positive reaction is defined as an induration ≥ 5 mm.

10.0 **STUDIES TO BE PERFORMED**

Each volunteer will receive up to four injections of vaccine at Months 0, 1, 6 and 12. The sections below describe the schedule to be observed for prescreening and on each inoculation day and afterwards. On the day of each immunization, all blood for laboratory evaluations must be drawn before the immunization.

10.1 Safety

Assessment of product safety (i.e., local and systemic reactions) will include extensive monitoring of hematologic and chemistry parameters.

The following sections provide a detailed listing of the safety and immunogenicity determinations and studies to be performed in this protocol at designated time points.

10.2 Plan for Evaluation of Immune Responses

Assays are performed on all volunteers at the time points indicated in Appendix E, unless otherwise noted.

The following parameters will be evaluated:

A. Humoral immune responses to HIV-1:
   1. Serum antibody binding reactivity (CIL)
      a. HIV EIA at AVEUs
      b. ELISA panel to gp160 MN and MN V3 peptides (CIL)
   2. Functional antibody activity (CIL)
      a. Neutralization titers against MN and RF (or isolates with similar sequences) and field HIV isolates
      b. Fusion inhibition titers
      c. Inhibition of gp120 binding to CD4

B. Cellular immune responses to HIV-1:
   1. T cell proliferative responses to PHA, Candida, T1-SP10 and SP10 peptides, and rgp160 MN. [Study Chair, cryopreserved cells]
   2. CTL assays [All volunteers, CIL] and at AVEU (U Washington volunteers) or at Beth Israel (Vanderbilt volunteers). To facilitate these studies, PBMC isolated pre-immunization from all subjects will be transformed by Epstein-Barr Virus (EBV) using procedures outlined in the AVEG *Manual of Operations*.

C. Ancillary Studies
   1. Binding to T1-SP10 peptides and to truncated isolate-specific peptides in RIA [Haynes Laboratory]
   2. Binding to HIV gp120 in RIP/Western blot or ELISA assays [Haynes Laboratory]
   3. LDA assays at Beth Israel (Vanderbilt volunteers)
   4. Assessment of cytokine induction [AVEUs]

10.3 Calendar of Studies (Refer to Appendix F)

DAYS -56 to -1 *PRESCREEN*

Preadmission screening tests and evaluations will be used to determine the eligibility of each volunteer for the study. All inclusion and exclusion criteria must be assessed within 8 weeks prior to study entry.

- Signed screening consent form
- Pre-study interview
- Medical history and complete physical examination (examination may be done on Day 0, prior to immunization)
- Counseling on prevention of HIV infection and pregnancy
- Negative pregnancy test (urine or serum) *[females, within 3 days prior to immunization]*
- Urine dipstick with esterase and nitrite
- Hepatitis B surface antigen
- RPR (syphilis serology)

  NOTE: If serology documented to be a false positive or due to a remote (>6 months) treated infection, the volunteer is eligible.
- Chest x-ray within 4 weeks prior to immunization
- Documented PPD skin test within one year

  NOTE: Volunteers with a positive PPD skin test who have a normal chest x-ray are eligible for participation in this trial, if there are no findings consistent with active pulmonary tuberculosis and no indications exist for isoniazid prophylaxis or treatment.
- ALT, GGT, and creatinine
- CBC, differential and platelet count
- T cell subsets
- HIV ELISA (Western Blot, if positive)
- 15 ml of heparinized blood for HLA typing (Class I only, AVEUs)
- 20 ml heparinized blood for EBV transformation of B cells at the AVEU (All volunteers, at least 2 weeks prior to vaccination)
- 20 ml heparinized blood shipped to CIL for parallel set up of EBV lines (All volunteers, at least 2 weeks prior to vaccination)
- 120 ml of ACD blood for lymphocyte proliferation, separation and cryopreservation of mononuclear cells for AVEU and long-term storage and for future HIV culture or PCR assays *[frozen for all volunteers; samples for lymphoproliferation shipped to Vanderbilt AVEU, when requested via memo from the DCAC; HIV culture or PCR to be performed at AVEU if HIV infection is suspected]*
- 50 ml of whole blood for serum for antibody studies, storage and distribution: [*CIL (6 ml), Repository storage (9 ml - including 3ml held for Wyeth-Lederle and shipped upon request), remaining vials stored at AVEU*]

DAY 0 *DAY OF FIRST IMMUNIZATION*

<u>Prior to Immunization</u>

- Signed protocol consent form
- Counseling on prevention of HIV infection and pregnancy
- Clinical evaluation including vital signs and symptoms assessment; specifically, volunteers should be asked about alcohol intake
- Negative pregnancy test [within 3 days prior to immunization]
- 30-70 ml of ACD blood [All volunteers, 30 ml shipped to CIL for cryopreservation for future assays; 40 ml for assays done at AVEU or Beth Israel]

IMMUNIZATION *[See Section 9.2.5]*

<u>Following Immunization</u>

NOTE: Subjects will be observed for 30 minutes post immunization. Temperatures and reactions will be assessed by the staff at the end of the ½ hour post vaccination observation period. Volunteers will take and record their temperatures the same evening (and at additional times if they have symptoms of fever) and record any other symptoms they experience.

DAYS 1-3

- Post-immunization follow-up

  NOTE: Volunteers will take and record temperature daily for 3 days following immunization and will communicate results to the AVEU. Clinical evaluation will be performed at the clinic on Days 1 or 2 post vaccination; subjects may be seen in the clinic or contacted by telephone on the other days. All volunteers having any reaction other than mild will be seen by the study staff within 24 hours.

DAY 7

- Study staff will contact volunteers by telephone for clinical evaluation.

DAY 28 *DAY OF SECOND IMMUNIZATION*

NOTE: Pregnancy test, immunization and immunization follow-up are <u>not required</u> for volunteers who had not completed the Day 28 visit by 3/10/98.

<u>Prior to Immunization</u>

NOTE: Studies must be performed prior to immunization, but it is not necessary to have results for any tests <u>except</u> the pregnancy test.

- Clinical evaluation, including vital signs and symptom-directed exam; specifically, volunteers should be asked about alcohol intake
- Counseling on prevention of HIV infection and pregnancy
- Urine dipstick with esterase and nitrite *[If abnormal, complete clean catch urinalysis is required]*
- Negative urine or serum pregnancy tests for females [within 3 days prior to immunization]
- ALT, GGT and creatinine
- CBC, differential and platelet count
- T cell subsets
- 90 ml of ACD blood for lymphocyte proliferation, mononuclear separation and cryopreservation for CMI studies for AVEUs and long-term storage *[frozen for all volunteers; samples for lymphoproliferation shipped to Vanderbilt AVEU, when requested via memo from the DCAC]*
- 50 ml of whole blood for serum for antibody studies, storage and distribution: *[CIL (3 ml), Repository storage (9 ml - including 3 ml held for Wyeth-Lederle and shipped upon request), remaining vials stored at AVEU.]*

IMMUNIZATION *[See Section 9.2.5]*

<u>Following immunization</u> *[See NOTE for DAY 0]*

DAYS 29-31

NOTE: Immunization follow-up procedures are <u>not required</u> for volunteers who had not completed the Day 28 visit by 3/10/98.

- Post-immunization follow-up *[See NOTE for DAYS 1 - 3]*

DAYS 35

NOTE: This visit is <u>not required</u> for volunteers who had not completed the Day 28 visit by 3/10/98.

- Study staff will contact volunteers by telephone for clinical evaluation.

DAY 42

NOTE: This visit is <u>required</u> for ALL volunteers.

- Clinical evaluation, including vital signs and symptom-directed exam
- Urine dipstick with esterase and nitrite *[If abnormal, complete clean catch urinalysis is required]*
- ALT, GGT and creatinine
- CBC, differential, platelet count
- T cell subsets
- 70 ml of ACD blood for CTL assays or ancillary cellular studies [All volunteers, 30 ml shipped to CIL; 40 ml for assays done at AVEUs or Beth Israel]
- 50 ml of whole blood for serum for antibody studies, storage and distribution:*[CIL (3 ml), Repository storage (9 ml - including 3ml held for Wyeth-Lederle and shipped upon request), remaining vials stored at AVEU*]

DAY 56

NOTE: This visit is <u>required</u> for ALL volunteers.

- Clinical evaluation, including vital signs and symptom-directed exam
- 90 ml of ACD blood for lymphocyte proliferation, mononuclear separation and cryopreservation for CMI studies for AVEUs and long-term storage *[frozen for all volunteers; samples for lymphoproliferation shipped to Vanderbilt AVEU, when requested via memo from the DCAC]*
- 50 ml of whole blood for serum for antibody studies, storage and distribution: [CIL (3 ml), Repository storage (9 ml - including 3ml held for Wyeth-Lederle and shipped upon request), remaining vials stored at AVEU]

DAY 196

NOTE: This visit is <u>required</u> for ALL volunteers.

- Clinical evaluation, including vital signs and symptom-directed exam
- Urine dipstick with esterase and nitrite *[If abnormal, complete clean catch urinalysis is required]*
- ALT, GGT and creatinine
- CBC, differential, platelet count
- T cell subsets
- 70 ml of ACD blood for CTL assays or ancillary cellular assays [All volunteers, 30 ml shipped to CIL; 40 ml for assays done at AVEUs or Beth Israel]

- 90 ml of ACD blood for lymphocyte proliferation, mononuclear separation and cryopreservation for CMI studies for AVEUs and long-term storage *[frozen for all volunteers; samples for lymphoproliferation shipped to Vanderbilt AVEU, when requested via memo from the DCAC]*
  NOTE: Previously required at Day 182.
- 50 ml of whole blood for serum for antibody studies, storage and distribution: *[CIL (3 ml), Repository storage (9 ml - including 3 ml held for Wyeth-Lederle and shipped upon request), remaining vials stored at AVEU.]*

DAY 378

NOTE: This visit is <u>required</u> for ALL volunteers. It is the final required protocol visit. Therefore, standard AVEG procedures for the final protocol visit will be performed at this visit instead of Day 532 (as scheduled in the original protocol).

- Complete physical examination (per standard AVEG procedure at final protocol visit)
- Chest x-ray
- Social Information Survey (per standard AVEG procedure at final protocol visit)
- Urine dipstick with esterase and nitrite *[If abnormal, complete clean catch urinalysis is required]*
- ALT, GGT and creatinine
- CBC, differential, platelet count
- T cell subsets
- HIV-1 ELISA and Western Blot (per standard AVEG procedure at final protocol visit)
- 70 ml of ACD blood for CTL assays or ancillary cellular assays [All volunteers, 30 ml shipped to CIL; 40 ml for assays done at AVEUs or Beth Israel]
- 90 ml of ACD blood for lymphocyte proliferation, mononuclear separation and cryopreservation for CMI studies for AVEUs and long-term storage *[frozen for all volunteers; samples for lymphoproliferation shipped to Vanderbilt AVEU, when requested via memo from the DCAC]*
  NOTE: Previously required at Day 364.
- 50 ml of whole blood for serum for antibody studies, storage and distribution: *[CIL (3 ml), Repository storage (9 ml - including 3 ml held for Wyeth-Lederle and shipped upon request), remaining vials stored at AVEU.]*
- DTH antigen placed *[Refer to Section 9.3 for instructions for preparation of dosing solutions, administration, and measurement of induration] (Previously required at Day 532.)*
  - Volunteers will be monitored in the clinic for 30 minutes after the skin tests are placed for signs of acute reaction, including a local wheal and flare.
  - 48 hours after DTH antigen placement (Day 380) volunteers will return to clinic for clinical evaluation, including vital signs and symptoms assessment, and evaluation of size of DTH responses

  NOTE: For Volunteers who complete the Day 378 visit before DTH reagents become available, the volunteer should be asked to return to the clinic for an additional visit for the DTH testing and the chest x-ray, if not done at Day 378.

NOTE: The remaining study visits (Days 392, 448 and 532 in the original protocol) are <u>not required</u>.

11.0 REFERENCES

1. *Human Retroviruses and AIDS 1991*. Myers G, Korber B, Berzofsky J, Smith RF, Pavlakes GN (eds). Los Alamos National Laboratory, Los Alamos, NM, 1991, pp III-6 to III-23.

2. La Rosa G, et al. *Science* 249:932-935, 1990.

3. Holley LH, et al. *Proc Natl Acad Sci (USA)* 88:6800-6804, 1991.

4. Jiang S, et al. *J Exp Med* 174:1557-1563, 1991.

5. Jahaverian K, et al. *Science* 250:1590-1593, 1990.

6. Haynes BF, et al. *AIDS Res Human Retroviral* 6:38-39, 1990.

7. Palker TJ, et al. *Proc Natl Acad Sci (USA)* 85:1932-1936, 1988.

8. Palker TJ, et al. *J Immunol* 142:3612-3619, 1989.

9. Hart MK, et al. *J Immunol* 145:2677-2685, 1990.

10. Hart MK, et al. *Proc Natl Acad Sci (USA)* 88:9448-9452, 1991.

11. Hart MK, and Haynes BF. Unpublished 1991.

12. Haynes BF, Arthur L et al. Conversion of an immunogenic HIV envelope synthetic peptide to a tolerogen in chimpanzees by the fusogenic domain of HIV gp41 envelope protein. *J Exp Med* 177:717-727, 1993.

13. Haynes BF, et al. Induction of cross-reactive HIV neutralizing antibodies in primates using a prime-boost regimen of hybrid synthetic gp120 envelopes peptides. *J Immunol* 151:1646-1653, 1993.

14. Methez D, et al. *Proc Natl Acad Sci (USA)* 87:7438-7442, 1990.

15. Karynas A, et al. *Proc Natl Acad Sci (USA)* 87:7613-7617, 1990.

16. Rusche J. et al. *Proc Natl Acad Sci (USA)* 95:3198-3203, 1988.

17. Safrit JT, Lee A, Andrews CA, Koup RA. A region of the third variable loop of HIV-1 gp120 is recognized by HLA-B7 restricted CTLs from two acute seroconversion patients. *J Immunol* 153:3822-3830, 1994.

18. Yasutomi Y, Palker TJ, Gardner MB, Haynes BF, Letvin NL. Synthetic peptide in mineral oil adjuvant elicits SIV-specific CD8+ CTL in rhesus monkeys. *J Immunol* 151:5096-5105, 1993.

19. Clerici M et al. *J Immunol* 146:2214-2219, 1991.

12.0 **INFORMED CONSENT PROCEDURES**

The informed consent process for AIDS vaccine trials is composed of two separate steps. The first step is used to secure the consent of a volunteer for screening tests and for obtaining information needed to determine the volunteer's eligibility for study participation. This initial consent form is not protocol specific and may be used separately by the institution to collect information from potential volunteers.

The second step is protocol-specific consent form, which describes the vaccine product to be used and all aspects involved in the protocol participation.

The following sample consent forms are provided on the following pages:

- Sample Consent Form for Participation in Pre-Trial Eligibility Tests
- Sample Consent Form for Participation in Protocol 020 - Version #2
- Sample Consent Form for Participation in Protocol 020 - Version #3 (Amendment I)

AIDS VACCINE EVALUATION GROUP

SAMPLE CONSENT FORM FOR PARTICIPATION IN PRE-TRIAL ELIGIBILITY TESTS

Principal Investigator ____ **Phone Number** ______________________________

INFORMED CONSENT

You are being asked to volunteer for blood tests and medical examinations to see if you are eligible for a vaccine research study against the human immunodeficiency virus (HIV). HIV is the virus that causes AIDS.

Before you can decide if you want to take part in the examinations and blood tests, you need to know the purpose of the screening, the possible risks and benefits and what is expected of you. Then, you can decide whether or not you want to have the tests. This process is called informed consent.

At the end of the tests, you will find out if you are eligible to be in the vaccine study. If you are eligible, the study staff will explain the vaccine study to you. If you decide to be in the vaccine study, you will sign another consent form.

YOUR PARTICIPATION IS VOLUNTARY

This consent form gives information about the screening tests. The study staff will discuss these tests with you and will answer any questions you have. If you agree to have the tests, you will be asked to sign this consent form and you will be given a copy to keep. You must know certain things:

- It is entirely up to you whether you have the tests.
- It is OK if you don't want to have the tests, or if you want to stop the tests. You can still take part in another research study later, if one is available.
- Even if you have the tests, you don’t have to join the vaccine research study.

PURPOSE OF THE TESTS

The purpose of the tests is to find out if you are eligible for a vaccine research study. Some people may not be able to take part in the vaccine study due to scientific or health reasons found during the tests and examinations.

PROCEDURES

Tests and examinations

The screening begins about 1-2 months before the start of the vaccine research study. During this time, you will come to the clinic at least two times, once to have a physical exam and a second time to receive the results of your tests. The study staff will also ask you some very personal questions about your sexual activity and drug use. As part of the physical exam, you will be asked to give a urine sample and a blood sample of about ½ to 1 cup. Your blood will be identified by

number only; your name will not be used. The blood and urine tests will include tests for HIV, Hepatitis B, liver function, and syphilis. You will also have a TB skin test and a chest x-ray.

A blood test will be done to see if you have HIV. If you are found to be HIV positive, you will not be able to enter this vaccine study. If the first blood test cannot tell for certain whether or not you are HIV positive, you will be offered more testing to find out whether you are really infected with HIV or if the test gave a false result. You will be referred for medical care if these tests show that you are infected with HIV. You may also be referred to another research study.

Women will be given a pregnancy test. If you are pregnant, you cannot be in the study because we do not know what effects HIV vaccines may have on the unborn fetus. If you are not pregnant, you will be asked to use an adequate method of birth control for one month before the vaccine study starts and for about 18 months after you begin the study.

All test results will be given to you and explained within one month of your exam.

POSSIBLE RISKS

Blood drawing may cause pain and bruising, and rarely, infection at the place where the blood is taken. Sometimes, drawing blood causes people to feel lightheaded or even faint. The questions about your sexual activity and drug use might be embarrassing.

If you decide to take part in the research vaccine study, you will be given an explanation of all the known risks.

POSSIBLE BENEFITS

The tests and examinations will not be of direct benefit to you. However, the information you receive about your blood tests and physical examination may be of some benefit to you.

CONFIDENTIALITY

Your research records will be confidential to the extent permitted by law. You will be identified by a code, and personal information from your records will not be released without your written permission. You will not be personally identified in any publication about this study. However, your records may be reviewed, under guidelines of the Federal Privacy Act, by the U.S. Food and Drug Administration; the National Institute of Allergy and Infectious Diseases, Division of AIDS; study monitors; and, pharmaceutical company(ies) that supply the study vaccines.

PUBLIC HEALTH RESPONSIBILITY

If the test results show that you are infected with HIV, the clinic staff will tell you as soon as possible. You will also receive the results of tests for other viruses and conditions that may affect your health. The project staff will help you find the right medical and/or public health people to give you advice. You will also be counseled about your responsibility to prevent the spread of HIV and other diseases to others.

REASONS FOR STOPPING THE SCREENING TESTS WITHOUT YOUR CONSENT

You may be removed from screening for the vaccine study without your consent for the following reasons:

- If your doctor feels that having the tests may be harmful to you
- If you don't keep appointments
- If the study sponsor decides to stop or cancel the study

COSTS TO YOU

You do not have to pay for the clinic visits and laboratory tests that are needed to see if you are eligible for the vaccine study. All other medical costs (including new tests ordered as a result of abnormal screening results) will be paid by you or your health insurance carrier, if you have insurance.

POLICY ABOUT RESEARCH RELATED INJURIES

If you are injured as a result of the prescreening, the ______________ (name of the clinic) will give you immediate necessary treatment for your injuries. The cost for this treatment will be charged to you or your insurance company. You will then be told where you may receive additional treatment for injuries. There is no program for monetary compensation or other forms of compensation for such injuries.

PROBLEMS OR QUESTIONS:

If you ever have questions about this study or if you believe you have had a research-related injury, you should contact (name of investigator) at (telephone number). If you have questions about your rights as a research subject, you can call (name and title of IRB member) at (telephone number).

SUBJECT'S CONSENT

If you have read this consent form (or if you have had it explained to you) and understand the information, and you voluntarily agree to take part in this study, please sign your name below.

______________________	______________________	______________
Volunteer's Name (typed or printed)	*Volunteer's Signature*	*Date*
______________________	______________________	______________
Witness' Name (typed or printed)	*Witness' Signature*	*Date*
______________________	______________________	______________
Investigator's Name (typed or printed)	*Investigator's Signature*	*Date*

AIDS VACCINE EVALUATION GROUP

SAMPLE CONSENT FORM FOR PARTICIPATION
in
A PHASE I SAFETY AND IMMUNOGENICITY TRIAL OF HIV-1 GP120 C4-V3
HYBRID POLYVALENT PEPTIDE IMMUNOGEN
MIXED IN MINERAL OIL CONTAINING MANNOSE MONO-OLEATE (IFA)
(Version 2)

Principal Investigator ________________________ Phone Number___________________

INFORMED CONSENT

You are being asked to take part in a research study to test an experimental vaccine that has been made to use against the human immunodeficiency virus (HIV). HIV is the virus that causes AIDS. Before you decide if you want to be in this study, you need to know the purpose of the study, the possible risks and benefits, and what is expected of you. Then, you can decide whether or not you want to take part in the study. This process is called informed consent.

YOUR PARTICIPATION IS VOLUNTARY

This consent form gives you information about the study. The clinic staff will discuss the information with you and will answer any questions you have. Once you understand the study, and if you agree to take part, you will be asked to sign this consent form. You will be given a copy to keep.

It is important that you know certain things:
- Your participation is entirely voluntary.
- You may decide not to take part or to withdraw from the study at any time without losing benefits you would normally have.

PURPOSE OF THE STUDY

The main purpose of this study is to see if this experimental vaccine is safe to take. Another important purpose is to study the responses that the immune system makes to the vaccine. A total of 28 people will take part in the study in three cities in the U.S. The study will last about 18 months.

Vaccines are given to people to try to resist or prevent a disease. The vaccine in this study is called C4-V3 peptide vaccine. The vaccine is a synthetic preparation of a small portion of the outer coat protein of HIV. It is mixed with an adjuvant called Incomplete Freund's Adjuvant (IFA). An adjuvant is a substance that increases the immune response.

The vaccine has been tested in mice, goats, monkeys and chimpanzees. The animal studies show that C4-V3 appears to be safe in doses planned for this study. The vaccine has also been tested as a vaccine in 10 patients who were infected with HIV. There were no bad reactions to the vaccine in that study.

It is important to remember that this vaccine has only a small portion of one protein from the HIV virus. Since it was <u>NOT</u> produced from live HIV virus or from an HIV-infected human cell line,

there is NO possibility that the vaccine contains live or killed HIV virus. Therefore, it is NOT possible that you will get HIV infection or AIDS by receiving the vaccine.

We do not know if this vaccine will provide any protection for you against HIV virus infection. Therefore, PLEASE DO NOT DO ANYTHING THAT MIGHT EXPOSE YOU TO HIV (SUCH AS UNPROTECTED SEX OR SHARING NEEDLES FOR INJECTION).

In this study, some people will receive the IFA adjuvant alone, without the C4-V3 as a placebo. Responses to the placebo are compared to responses to the C4-V3 plus IFA.

You should be aware that a few people who received experimental HIV vaccines or placebos in the past became infected with HIV through sex or drug use. We know that HIV infection and AIDS can develop even in people who received test vaccine if they are exposed to HIV. If you are exposed to HIV after receiving this vaccine, your risk of becoming infected with HIV and developing AIDS is unknown. It is possible that the vaccine: 1) will protect you against HIV infection, 2) will not protect you against HIV infection, or 3) make you more likely to become HIV infected. If you do become infected, we do not know what effect the vaccine may have on the disease. The time that it takes for you to become sick from HIV/AIDS may be the same, longer or even shorter than expected. PLEASE DO NOT DO ANYTHING THAT MIGHT EXPOSE YOU TO HIV (SUCH AS HAVING UNPROTECTED SEX OR SHARING NEEDLES FOR INJECTION). You will be educated and counseled about HIV exposure during the study. If you have questions, please ask the clinic staff.

STUDY PROCEDURES

Entering the Study. Before entering the study, you will have a physical examination and blood and urine tests. You will have a chest x-ray. Women will have a urine pregnancy test. There will also be an interview about your health and you will be asked questions about your sexual activity and drug use.

Study Groups. If you agree to join the trial, you will be assigned to one of 3 study groups. The groups are assigned by randomization (a computer process that is much like tossing a coin). Neither you nor the clinic staff will know if you receive C4-V3 plus IFA or IFA alone until the end of the study.

The study groups are:
- Lower dose of C4-V3 mixed with IFA
- A higher dose of C4-V3 mixed with IFA
- IFA alone

The responses of the people who receive IFA alone will be compared to those who receive C4-V3.

You will have vaccine shots at four times during the study. Each time you will have 2 shots -- one in each arm. The first shots will be at the beginning of the study, and then again after 1 month, 6 months and 12 months.

Vaccine Shots and Follow-up. The vaccine shots will be given into arm muscle (one shot into each arm). You must stay at the clinic for 30 minutes after each shot so the clinic staff can watch for any reaction. You will be asked to record your temperature and symptoms each day for 3 days and report them to the clinic staff. You will be asked to come to the clinic on the first or second day after each shot. If you have a reaction, you may be asked to come into the clinic at other

times to be checked. You will be asked to report any side effects to one of the study physicians or nurses right away.
<u>Length of the Study</u>. The study will last about 18 months. During the study, you will need to have laboratory tests and physical examinations. This means that you will need to come to the clinic for about 15 visits to have these tests done. The visits will usually take no more than 1 hour. During the study, you will be asked to limit the amount of alcohol you drink to no more than one drink (1 ounce of alcohol) each day. It is very important that you follow the instructions given to you by the medical staff.

When the study is completed, with your permission, the study staff will contact you once or twice a year for at least five more years to check on your health. If any new information is discovered about effects this vaccine might have on your health, the study staff will need to contact you.

<u>Tests Done During the Study</u>. The total amount of blood that you will give during the study will not be more than about 8 cups. You might also be asked to have laboratory tests between regular visits. Some of your blood will be stored with the usual protectors of identity. This blood may be used for AVEG-approved AIDS-related research in the future.

<u>Skin Tests</u>. At 18 months, you will have a skin test done. For the skin test (called the HIV-1 C4-V3 skin test), a small amount of the protein in the vaccine is injected under the skin on your forearm (the inside of your arm, below the elbow). Two different doses of the skin test will be tested. If your immune system “remembers” the vaccine protein, it will respond in 2-3 days. A localized inflammation (redness and swelling) at the injection site will result. Two commonly used skin tests will be used for comparison. Tetanus toxoid is an FDA-approved vaccine that is often used for skin testing. Candida (a common yeast) is FDA-approved for the type of skin testing planned in this study. Most people have been exposed to or vaccinated with these, and should have a positive skin test. The response to these skin tests will be compared to the response to the HIV-1 C4-V3 skin test. Also, the saline (salt) solution (used to mix the HIV-1 C4-V3) will be injected alone (placebo control). You will have a total of 5 injections for the skin testing. You will come to the clinic 2 days later. The study clinician will examine the skin test site and measure any redness or swelling.

<u>Monitoring the Study</u>. The study will be monitored by a group of experts known as the AIDS Vaccine Data and Safety Monitoring Board (AVDSMB). This group will review the information from the study. They will pay close attention to harmful reactions. If the AVDSMB decides that significant adverse events have occurred, further injections may be delayed or canceled.

POTENTIAL RISKS

<u>Vaccine</u>. The possible risks for this vaccine include fever, chills, rash, aches and pains, nausea, headache and fatigue. We know these side effects can occur with other vaccines. They can occur whether you receive the test vaccine or placebo in this study. If you do have a reaction, this does not mean you received the active vaccine. The side effects don't usually last long and people usually don't need to be treated for them.

As with all vaccines or drugs, you could have an allergic reaction—a rash, hives or even difficulty breathing. Allergic reactions can be dangerous; therefore, the clinic staff will watch you for 30 minutes after each shot. There may be other side effects, even serious ones, that we don't know about yet. Therefore, it is important that you report any side effects to the clinic staff as soon as they occur.

Historically, some vaccines using IFA as an adjuvant have caused severe local reactions; this varies with the type of vaccine and the type of IFA. In this study, IFA is used because it gave the best immune response to the vaccine in animal testing. The IFA for this study was chosen because it is less likely to cause reactions than older brands. In DATRI 010 (the only other study of this product in humans), 50% of persons had a slight soreness at the injection site, and one person out of 10 had fever and chills that lasted less than 48 hours.

Skin Tests. The possible side effects of the skin tests include pain, soreness, redness, swelling, and itching at the injection site. Skin tests don't usually cause symptoms such as fevers, chills, headaches, nausea, fatigue or swollen lymph nodes; however, these symptoms are sometimes seen when people have larger doses of this vaccine injected into their arm muscles. Tetanus and Candida (yeast) skin test antigens have been used in many people without causing any significant side effects. There may be side effects related the HIV-1 C4-V3 skin test that we don't know about yet.

Blood Drawing and Vaccine Shots. Blood drawing may cause pain and bruising, and rarely, infection at the site where the blood is taken. Sometimes, drawing blood causes people to feel lightheaded or even faint. Injections into a muscle can cause pain, soreness, redness and swelling.

Pregnancy. We do not know the possible effects of the vaccine on the unborn fetus. Therefore, women must have a negative pregnancy test before each vaccine shot. They must also agree to practice birth control during the study or be known to be unable to have children. If it is determined that you are pregnant during the study, you will be given no further injections; however, you will be asked to continue study follow-up visits.

Other Risks. Taking the test vaccine might mean that you will not be able to take other experimental AIDS vaccines later. The effectiveness of these vaccines is not known. Therefore, **IT IS VERY IMPORTANT THAT YOU AVOID ANY HIGHER-RISK BEHAVIOR THAT WOULD PUT YOU AT RISK FOR BECOMING INFECTED WITH HIV**.

It is also possible that the vaccines could change how quickly your body develops AIDS if you should become infected with HIV after receiving the test vaccines. These vaccines may slow the speed of developing AIDS or they may lead to a more rapid onset of AIDS. They may also have no effect on the HIV related disease activity.

After the vaccine injections, you may test positive for HIV (by the ELISA test). However, it should be possible, by using other tests to show that your positive result on the HIV test is NOT because you are infected with HIV, but because you have received the test vaccine.

If you do have a positive HIV test caused by the experimental vaccine, we do not know how long the test may be positive. YOU MAY BE SUBJECTED TO THE SOCIAL RISKS OF "APPEARING" TO BE HIV-1 POSITIVE. You will not be able to donate blood. You may also have difficulties with:

- Getting insurance
- Hospitalization
- Traveling to other countries
- Employment
- Military service/Peace Corps
- Relationships with friends and family

If you have problems like these, help is available from the clinic staff and the NIH. To help avoid these problems, you will be offered an identification card that shows you joined this study. A toll-free number is listed on your card.

POSSIBLE BENEFITS

You will receive no direct benefits from taking part in this study. However, you and others may benefit in the future from the information that will be learned from the study.

NEW FINDINGS

You will be told of any new information learned during the course of the study that might cause you to change your mind about staying in the study. At the end of the study, you will be told when study results may be available and how to learn about them.

CONFIDENTIALITY OF RECORDS

Your research records will be confidential to the extent permitted by law. You will be identified by a code, and personal information from your records will not be released without your written permission. You will not be personally identified in any publication about this study. However, your records may be reviewed, under guidelines of the Federal Privacy Act, by the U.S. Food and Drug Administration; the National Institute of Allergy and Infectious Diseases, Division of AIDS; study monitors; and, pharmaceutical company that supplies the study vaccine.

PUBLIC HEALTH RESPONSIBILITY

You will be tested for HIV every 3-6 months during the study. At any time, if test results show that you are infected with the HIV virus, the clinic staff will tell you, in person, as soon as possible. They will help you find the right medical and/or public health people to give you advice. They will also help you carry out your responsibility to contact anyone with whom you had sex or shared needles. It is important that these people find out that they may have been exposed to HIV. They should be tested as soon as possible. The clinic staff will also advise you and your sexual partner(s) about the meaning of the test results and how to avoid passing the virus to other people.

REASONS WHY YOU MAY BE WITHDRAWN FROM THE STUDY WITHOUT YOUR CONSENT

You may be withdrawn from the study without your consent for the following reasons:

- If the investigator or your private doctor feels that staying in the study would be harmful to you
- If you don't keep appointments or follow study procedures
- If you have serious side effects from the vaccine
- If the study sponsor or vaccine manufacturer or the FDA decides to stop or cancel the study
- If the Data and Safety Monitoring Board feels that the study should be stopped

If you agree to take part in this study, it is important for you to keep all your appointments. However, if you don't want to stay in the study, you can leave at any time. You will not lose any benefits that you would have if you had not joined the study.

COSTS TO YOU

You do not have to pay for the vaccine, research clinic visits, examinations or laboratory tests that are part of this study. All other medical costs outside this study will be paid by you or your health insurance carrier (if you have insurance). You will be given $_____ for each visit that you complete. The money will be given to you ___ times throughout the study. $ _____ will be given to you each time.

RESEARCH- RELATED INJURY

If you are injured as a result of being in this study, the ____________________ (name of the clinic) will give you immediate necessary treatment for your injuries. The cost for this treatment will be charged to you or your insurance company. You will then be told where you may receive additional treatment for injuries. There is no program for monetary compensation or other forms of compensation for such injuries.

PROBLEMS OR QUESTIONS

If you ever have questions about this study or in case of research-related injuries, you should contact (name of investigator) at telephone number _____________. If you have questions about your rights as a research subject, you can call ____________ (IRB member) at telephone number ________________________.

SUBJECT'S CONSENT

If you have read this consent form (or if you have had it explained to you) and understand the information, and you voluntarily agree to take part in this study, please sign your name below.

I agree to join in this research study.

________________________ ___________________________ ______________

Volunteer's Name *Volunteer's Signature* *Date*
(typed or printed)

________________________ ___________________________ ______________

Witness' Name *Witness' Signature* *Date*
(typed or printed)

________________________ ___________________________ ______________

Investigator's Name *Investigator's Signature* *Date*
(typed or printed)

AIDS VACCINE EVALUATION GROUP

SAMPLE CONSENT FORM FOR PARTICIPATION
in
A PHASE I SAFETY AND IMMUNOGENICITY TRIAL OF HIV-1 GP120 C4-V3 HYBRID POLYVALENT PEPTIDE IMMUNOGEN MIXED IN MINERAL OIL CONTAINING MANNOSE MONO-OLEATE (IFA)
(Version 3)

AMENDMENT I: Chest X-ray at last study visit

Principal Investigator ______________________ **Phone Number**___________________

INFORMED CONSENT

You are being asked to agree to have a chest x-ray done at your last study visit. This x-ray is being done to make sure there have been no changes since the chest x-ray you had before the study began. A chest x-ray exposes you to a small amount of radiation. This amount is far below what a normal person is exposed to from the environment over one week.

If you agree to have the additional x-ray, you will be asked to sign this consent form. You will be given a copy to keep. It is important that you know the following:

- Your participation is entirely voluntary
- You may decide not to have the additional chest x-ray without losing benefits you would normally have.

Other Information

The following sections of the consent form you signed when you joined the study also apply to this amendment:

- **POSSIBLE BENEFITS**
- **CONFIDENTIALITY OF RECORDS**
- **COSTS TO YOU FOR YOUR PARTICIPATION**
- **POLICY ABOUT RESEARCH RELATED INJURIES**
- **PROBLEMS OR QUESTIONS:**

Subject's Consent

If you have read this consent form (or if you have had it explained to you) and agree to take part in this amended study, please sign your name below.

____________________	____________________	______________
Volunteer's Name (typed or printed)	*Volunteer's Signature*	*Date*
____________________	____________________	______________
Witness' Name (typed or printed)	*Witness' Signature*	*Date*
____________________	____________________	______________
Investigator's Name (typed or printed)	*Investigator's Signature*	*Date*

13.0 MODIFICATIONS TO PROTOCOL

The following table describes the history of modifications to the original version of Protocol 020.

DATE	DOCUMENT	PROTOCOL MODIFICATION	COMMENT
07/13/97	Version 1	Original protocol	Version revised prior to FDA/IRB submission; see Version 2 below.
08/08/97	Version 2	Product designation changed to reflect name used in latest Investigator's Drug Brochure...<br>Necessitated changes to following pages:<br>- Title page<br>- Protocol summary page<br>- First paragraph on p. 10 - Section 6.0<br>- Consent Form title on p.30	
3/20/98		Clarification #1	Revised instructions for serum distribution
4/20/98		Clarification #2	Modify visit schedule in response to suspended enrollment and immunization
12/14/98	Version 3	Amendment I	Add chest x-ray to final visit; revise instructions for DTH schedule

AIDS VACCINE EVALUATION GROUP

CLARIFICATION #1

PROTOCOL 020: A PHASE I SAFETY AND IMMUNOGENICITY TRIAL OF HIV-1 GP120 C4-V3 HYBRID POLYVALENT PEPTIDE IMMUNOGEN MIXED IN MINERAL OIL CONTAINING MANNOSE MONO-OLEATE (IFA)

March 20, 1998
[BB IND #7289 - held by DAIDS]

SUMMARY OF REVISIONS

- Revise instructions for distribution of serum to the Repository, CIL and Sponsor

IMPLEMENTATION

The following modifications, indicated by redline/strikeout, are made to Protocol 020:

- Section 10.3, Calendar of Studies

  - Modify: Instructions for distribution of serum on Days -56 to -1, 28, 42, 56, 112, 182, 196, 210, 294, 364, 378, 392, 448, 532:
  - 50 ml of whole blood for serum for antibody studies, storage and distribution: [~~*CIL (6 ml), Repository (6 ml), Wyeth-Lederle (3* ml)~~] *[CIL (3 ml), Repository storage (9 ml - including 3 ml held for Wyeth-Lederle and shipped upon request), remaining vials stored at AVEU]*

AIDS VACCINE EVALUATION GROUP

CLARIFICATION #2

PROTOCOL 020:A PHASE I SAFETY AND IMMUNOGENICITY TRIAL OF HIV-1 GP120 C4-V3 HYBRID POLYVALENT PEPTIDE IMMUNOGEN MIXED IN MINERAL OIL CONTAINING MANNOSE MONO-OLEATE (IFA)

April 15, 1998
[BB IND #7289 - held by DAIDS]

SUMMARY OF REVISIONS

- The protocol visit schedule is simplified in response to the Protocol Team's decision of March 10, 1998 to suspend enrollment and further immunizations in the study. The final follow-up visit, including DTH testing, social impact survey and a physical exam, will occur at Day 378.

BACKGROUND

On March 10, 1998, the Protocol Team reviewed Serious Adverse Experience reports and verbal reports from the AVEUs. Several subjects in Protocol 020 have experienced either local or systemic reactions that are considered unacceptable (severe or persistent reactions at injection site or severe flu-like symptoms). The Protocol Team agreed that all further enrollment would be suspended and no further immunizations would be given to volunteers already enrolled. However, volunteers will be followed clinically to monitor volunteer safety, as needed, and blood draws will be continued, on a simplified schedule, to the Day 378 time point. A letter to volunteers was developed and made available to the AVEUs for distribution to participating volunteers.

IMPLEMENTATION

The following modifications, indicated by redline/strikeout, are made to the visit schedule (Section 10.3, Calendar of Studies).

CLARIFICATION #2 - continued

DAY 28 DAY OF SECOND IMMUNIZATION

NOTE: Pregnancy test, immunization and immunization follow-up are not required for volunteers who had not completed the Day 28 visit by 3/10/98.

Prior to Immunization

NOTE: Studies must be performed prior to immunization, but it is not necessary to have results for any tests except the pregnancy test.

- Clinical evaluation, including vital signs and symptom-directed exam; specifically, volunteers should be asked about alcohol intake
- Counseling on prevention of HIV infection and pregnancy
- Urine dipstick with esterase and nitrite *[If abnormal, complete clean catch urinalysis is required]*
- Negative urine or serum pregnancy tests for females [within 3 days prior to immunization]
- ALT, GGT and creatinine
- CBC, differential and platelet count
- T cell subsets
- 90 ml of ACD blood for lymphocyte proliferation, mononuclear separation and cryopreservation for CMI studies for AVEUs and long-term storage *[frozen for all volunteers; samples for lymphoproliferation shipped to Vanderbilt AVEU, when requested via memo from the DCAC]*
- 50 ml of whole blood for serum for antibody studies, storage and distribution: *[CIL (3 ml), Repository storage (9 ml - including 3 ml held for Wyeth-Lederle and shipped upon request), remaining vials stored at AVEU.]*

IMMUNIZATION *[See Section 9.2.5]*

Following immunization *[See NOTE for DAY 0]*

DAYS 29-31

NOTE: Immunization follow-up procedures are not required for volunteers who had not completed the Day 28 visit by 3/10/98.

- Post-immunization follow-up *[See NOTE for DAYS 1 - 3]*

DAYS 35

NOTE: This visit is not required for volunteers who had not completed the Day 28 visit by 3/10/98.

- Study staff will contact volunteers by telephone for clinical evaluation.

DAY 42

NOTE: This visit is required for ALL volunteers.

- Clinical evaluation, including vital signs and symptom-directed exam
- Urine dipstick with esterase and nitrite *[If abnormal, complete clean catch urinalysis is required]*
- ALT, GGT and creatinine
- CBC, differential, platelet count
- T cell subsets

CLARIFICATION #2 - continued

- 70 ml of ACD blood for CTL assays or ancillary cellular studies [All volunteers, 30 ml shipped to CIL; 40 ml for assays done at AVEUs or Beth Israel]

- 50 ml of whole blood for serum for antibody studies, storage and distribution:*[CIL (3 ml), Repository storage (9 ml - including 3 ml held for Wyeth-Lederle and shipped upon request), remaining vials stored at AVEU.]*

DAY 56

NOTE: This visit is required for ALL volunteers.

- Clinical evaluation, including vital signs and symptom-directed exam
- 90 ml of ACD blood for lymphocyte proliferation, mononuclear separation and cryopreservation for CMI studies for AVEUs and long-term storage *[frozen for all volunteers; samples for lymphoproliferation shipped to Vanderbilt AVEU, when requested via memo from the DCAC]*
- 50 ml of whole blood for serum for antibody studies, storage and distribution: *[CIL (3 ml), Repository storage (9 ml - including 3 ml held for Wyeth-Lederle and shipped upon request), remaining vials stored at AVEU.]*

DAY 112

NOTE: This visit is not required.

- ~~Clinical evaluation, including vital signs and symptom-directed exam~~
- ~~50 ml of whole blood for serum for antibody studies, storage and distribution: *[CIL (3 ml), Repository storage (9 ml - including 3 ml held for Wyeth-Lederle and shipped upon request), remaining vials stored at AVEU.]*~~

DAY 182 ~~*DAY OF THIRD IMMUNIZATION*~~

NOTE: The immunization originally scheduled for this visit is canceled; this visit is not required.

~~Prior to Immunization *[See NOTE for DAY 28]*~~

- ~~Clinical evaluation, including vital signs and symptom-directed exam; specifically, volunteers should be asked about alcohol intake~~
- ~~Counseling on prevention of HIV infection and pregnancy~~
- ~~Urine dipstick with esterase and nitrite *[If abnormal, complete clean catch urinalysis is required]*~~
- ~~Negative urine or serum pregnancy tests for females [within 3 days prior to immunization]~~
- ~~ALT, GGT and creatinine~~
- ~~CBC, differential and platelet count~~
- ~~T cell subsets~~
- ~~HIV ELISA (Western Blot, if positive)~~
- ~~90 ml of ACD blood for lymphocyte proliferation, mononuclear separation and cryopreservation for CMI studies for AVEUs and long-term storage *[frozen for all volunteers; samples for lymphoproliferation shipped to Vanderbilt AVEU, when requested via memo from the DCAC]*~~
- ~~50 ml of whole blood for serum for antibody studies, storage and distribution:*[CIL (3 ml), Repository storage (9 ml - including 3 ml held for Wyeth-Lederle and shipped upon request), remaining vials stored at AVEU.]*~~

CLARIFICATION #2 - continued

- ~~IMMUNIZATION *[See Section 9.2.5]*~~

~~Post-immunization follow-up *[See NOTE for DAY 0]*~~

DAYS 183-185
NOTE: This visit is not required.

- ~~Post-immunization follow-up *[See NOTE for DAYS 1 - 3]*~~

DAY 189
NOTE: This visit is not required.
- ~~Study staff will contact volunteers by telephone for clinical evaluation.~~

DAY 196
NOTE: This visit is required for ALL volunteers.
- Clinical evaluation, including vital signs and symptom-directed exam
- Urine dipstick with esterase and nitrite *[If abnormal, complete clean catch urinalysis is required]*
- ALT, GGT and creatinine
- CBC, differential, platelet count
- T cell subsets
- 70 ml of ACD blood for CTL assays or ancillary cellular assays [All volunteers, 30 ml shipped to CIL; 40 ml for assays done at AVEUs or Beth Israel]
- 90 ml of ACD blood for lymphocyte proliferation, mononuclear separation and cryopreservation for CMI studies for AVEUs and long-term storage *[frozen for all volunteers; samples for lymphoproliferation shipped to Vanderbilt AVEU, when requested via memo from the DCAC]*
  NOTE: Previously required at Day 182.
- 50 ml of whole blood for serum for antibody studies, storage and distribution: *[CIL (3 ml), Repository storage (9 ml - including 3 ml held for Wyeth-Lederle and shipped upon request), remaining vials stored at AVEU.]*

DAY 210
NOTE: This visit is not required.
- ~~Clinical evaluation, including vital signs and symptom-directed exam~~
- ~~90 ml of ACD blood for lymphocyte proliferation, mononuclear separation and cryopreservation for CMI studies for AVEUs and long-term storage *[frozen for all volunteers; samples for lymphoproliferation shipped to Vanderbilt AVEU, when requested via memo from the DCAC]*~~
- ~~50 ml of whole blood for serum for antibody studies, storage and distribution: *[CIL (3 ml), Repository storage (9 ml - including 3 ml held for Wyeth-Lederle and shipped upon request), remaining vials stored at AVEU.]*~~

DAY 294
NOTE: This visit is not required.
- ~~Clinical evaluation, including vital signs and symptom-directed exam~~
- ~~40 ml of ACD blood for ancillary CTL or other cellular assays [AVEUs or Beth Israel]~~

CLARIFICATION #2 - continued

- ~~50 ml of whole blood for serum for antibody studies, storage and distribution: *[CIL (3 ml), Repository storage (9 ml - including 3 ml held for Wyeth-Lederle and shipped upon request), remaining vials stored at AVEU.]*~~

DAY 364 ~~*DAY OF FOURTH IMMUNIZATION*~~

NOTE: The immunization originally scheduled for this visit is canceled; this visit is not required.

~~Prior to Immunization *[See NOTE for DAY 28]*~~

~~*NOTE: Studies must be performed prior to immunization, but it is not necessary to have results for any tests except the pregnancy test.*~~

- ~~Clinical evaluation, including vital signs and symptoms assessment; specifically, volunteers should be asked about alcohol intake~~
- ~~Counseling on prevention of HIV infection and pregnancy~~
- ~~Urine dipstick with esterase and nitrite *[If abnormal, complete clean catch urinalysis is required]*~~
- ~~Negative urine or serum pregnancy tests for females [within 3 days prior to immunization]~~
- ~~ALT, GGT and creatinine~~
- ~~CBC, differential and platelet count~~
- ~~T cell subsets~~
- ~~HIV ELISA (Western Blot, if positive)~~
- ~~90 ml of ACD blood for lymphocyte proliferation, mononuclear separation and cryopreservation for CMI studies for AVEUs and long-term storage *[frozen for all volunteers; samples for lymphoproliferation shipped to Vanderbilt AVEU, when requested via memo from the DCAC]*~~
- ~~50 ml of whole blood for serum for antibody studies, storage and distribution: *[CIL (3 ml), Repository storage (9 ml - including 3 ml held for Wyeth-Lederle and shipped upon request), remaining vials stored at AVEU.]*~~
- ~~IMMUNIZATION *[See Section 9.2.5]*~~

~~Following immunization [See NOTE for DAY 0]~~

DAYS 365-367

NOTE: This visit is not required.

- ~~Post-immunization follow-up. *[See NOTE for DAYS 1 - 3]*~~

DAY 371

NOTE: This visit is not required.

- ~~Study staff will contact volunteers by telephone for clinical evaluation.~~

DAY 378

NOTE: This visit is required for ALL volunteers. It is the final required protocol visit. Therefore, standard AVEG procedures for the final protocol visit will be performed at this visit instead of Day 532.

- ~~Clinical evaluation, including vital signs and symptom-directed exam~~
- Complete physical examination (per standard AVEG procedure at final protocol visit)

CLARIFICATION #2 - continued

- Social Information Survey (per standard AVEG procedure at final protocol visit)

- Urine dipstick with esterase and nitrite *[If abnormal, complete clean catch urinalysis is required]*
- ALT, GGT and creatinine
- CBC, differential, platelet count
- T cell subsets
- HIV-1 ELISA and Western Blot (per standard AVEG procedure at final protocol visit)
- 70 ml of ACD blood for CTL assays or ancillary cellular assays [All volunteers, 30 ml shipped to CIL; 40 ml for assays done at AVEUs or Beth Israel]
- 90 ml of ACD blood for lymphocyte proliferation, mononuclear separation and cryopreservation for CMI studies for AVEUs and long-term storage *[frozen for all volunteers; samples for lymphoproliferation shipped to Vanderbilt AVEU, when requested via memo from the DCAC]*
  NOTE: Previously required at Day 364.

- 50 ml of whole blood for serum for antibody studies, storage and distribution: *[CIL (3 ml), Repository storage (9 ml - including 3 ml held for Wyeth-Lederle and shipped upon request), remaining vials stored at AVEU.]*
- DTH antigen placed *[Refer to Section 9.3 for instructions for preparation of dosing solutions, administration, and measurement of induration] (Previously required at Day 532.)*
  - Volunteers will be monitored in the clinic for 30 minutes after the skin tests are placed for signs of acute reaction, including a local wheal and flare.
  - 48 hours after DTH antigen placement (Day 380) volunteers will return to clinic for clinical evaluation, including vital signs and symptoms assessment, and evaluation of size of DTH responses

NOTE: The remaining study visits (Days 392, 448 and 532) are not required.

DAY 392

- ~~Clinical evaluation, including vital signs and symptom-directed exam~~
- ~~90 ml of ACD blood for lymphocyte proliferation, mononuclear separation and cryopreservation for CMI studies for AVEUs and long-term storage *[frozen for all volunteers; samples for lymphoproliferation shipped to Vanderbilt AVEU, when requested via memo from the DCAC]*~~
- ~~50 ml of whole blood for serum for antibody studies, storage and distribution: *[CIL (3 ml), Repository storage (9 ml - including 3 ml held for Wyeth-Lederle and shipped upon request), remaining vials stored at AVEU.]*~~

DAY 448

- ~~Clinical evaluation, including vital signs and symptom-directed exam~~
- ~~40-70 ml of ACD blood for CTL assays, limiting dilution assays, and other ancillary cellular assays [Ancillary, 30 ml shipped to CIL and 40 ml for assays done at AVEUs or Beth Israel]~~
- ~~50 ml of whole blood for serum for antibody studies, storage and distribution: *[CIL (3 ml), Repository storage (9 ml - including 3 ml held for Wyeth-Lederle and shipped upon request), remaining vials stored at AVEU.]*~~

CLARIFICATION #2 - continued

DAY 532

- ~~Complete physical examination~~

- ~~Urine dipstick with esterase and nitrite *[If abnormal, complete clean catch urinalysis is required]*~~
- ~~ALT, GGT and creatinine~~
- ~~CBC, differential, and platelet count~~
- ~~T cell subsets~~
- ~~HIV-1 ELISA and Western Blot~~
- ~~90 ml of ACD blood for lymphocyte proliferation, mononuclear separation and cryopreservation for CMI studies for AVEUs and long-term storage *[frozen for all volunteers; samples for lymphoproliferation shipped to Vanderbilt AVEU, when requested via memo from the DCAC]*~~
- ~~50 ml of whole blood for serum for antibody studies, storage and distribution: *[CIL (3 ml), Repository storage (9 ml - including 3 ml held for Wyeth-Lederle and shipped upon request), remaining vials stored at AVEU.]*~~

AIDS VACCINE EVALUATION GROUP

AMENDMENT I

PROTOCOL 020: A PHASE I SAFETY AND IMMUNOGENICITY TRIAL OF HIV-1 GP120 C4-V3 HYBRID POLYVALENT PEPTIDE IMMUNOGEN MIXED IN MINERAL OIL CONTAINING MANNOSE MONO-OLEATE (IFA)

December 14, 1998
[BB IND #7289 - held by DAIDS]

SUMMARY OF REVISIONS

- The directions for the final protocol visit are modified to include a chest x-ray in the final physical examination.
- For volunteers who complete the Day 378 visit before DTH reagents become available, the volunteer should be asked to return to the clinic for an additional visit for the DTH testing and the chest x-ray, if not done at Day 378.

IMPLEMENTATION

The modifications indicated above are made to the visit schedule for Day 378 and incorporated into the text of Version 3 of the protocol, along with the modifications included in Clarifications #1 and #2. A detailed list of protocol modifications is available.

APPENDIX A

AIDS VACCINE EVALUATION GROUP
DEFINITION OF RISK CATEGORIES

I. DEFINITION OF TERMS

A. The only *absolute* way to prevent infection or transmission of HIV through sexual contact is to not have oral, anal, or vaginal intercourse with persons potentially infected (i.e., sex with self or no sex).

B. *SAFEST SEX*: mutual masturbation, kissing, body rubbing (frottage) erotic conversation (e.g., phone sex), unshared sex toys (wash hands before and after sex).

C. *POSSIBLY SAFE SEX*: condoms* for oral, anal and vaginal sex. Condoms should be latex only, put on before penetration.

D. *UNSAFE SEX*: oral, vaginal, or anal intercourse without condom*; vaginal, anal sex without condom where partner withdraws before ejaculation; fisting, rimming, shared sex toys.

E. For purposes of AVEG trials, we will define unprotected oral intercourse with an HIV+ partner as unsafe sex, with an unknown partner as possibly safe sex.

F. *HIGHER RISK-ASSOCIATED STD*: The following STDs are considered associated with higher risk sexual behavior:
- Neisseria gonorrhea
- Non-gonococcal urethritis
- Chlamydia trachomatis
- Newly acquired infection with herpes simplex virus, Treponema *pallidum* (syphilis), Trichomonas *vaginalis*
- Newly acquired mucopurulent cervicitis, acute epididymitis, acute proctitis
- Pelvic inflammatory disease
- Lymphogranuloma venereum
- Chancroid
- Sexually acquired Hepatitis B

The following STDs are not considered associated with higher risk sexual behavior:
- Scabies
- Yeast
- Pediculosis pubis
- Bacterial vaginosis
- Anogenital warts

* Substitute dental dam or equivalent in cases of oral-genital sex

APPENDIX A - continued

II. **RISK CATEGORIES**

RISK DEFINITION A:

EITHER ALL OF THE FOLLOWING

1. No newly acquired higher risk associated STD in the last 6 months
2. No possibly safe or unsafe sex with a known HIV+ individual or an active injection drug user in the past 6 months
3. No unsafe sexual activity
4. Possibly safe sexual activity with <u>two or fewer</u> partners within the last 6 months
5. No injection drug use

OR BOTH OF THE FOLLOWING

6. Mutually monogamous relationship with a known or presumed HIV seronegative partner for the last 6 months
7. No injection drug use

RISK DEFINITION B:

ALL OF THE FOLLOWING

1. No newly acquired higher risk associated STD in the last 6 months
2. No possibly safe or unsafe sex with a known HIV+ individual or an active injection drug user in the past 6 months
3. Unsafe sexual activity with <u>two or fewer</u> partners within the last 6 months
4. Possibly safe sexual activity with <u>four or fewer</u> partners within the last 6 months
5. No injection drug use in the last 1 year

RISK DEFINITION D:

ONE OR MORE OF THE FOLLOWING

1. A newly acquired higher risk-associated STD within the past 6 months.
2. Unsafe sexual activity with 4 or more partners with in the past 6 months
3. Possibly safe or unsafe sex with a known HIV+ individual in the past 6 months.
4. Possibly safe sexual activity with 12 or more partners in the past 6 months.
5. Any injection drug use in the last 1 year

RISK DEFINITION C:

ALL REMAINING INDIVIDUALS

APPENDIX B

SCREENING PROCEDURES FOR HIV VACCINE TRIAL VOLUNTEERS

Screening procedures for candidates for HIV trials include completion of a questionnaire that consists of a profile cover sheet and a general screening section. Subsequent to the screening interview, investigators will proceed with phlebotomy, medical history and physical examination.

A copy of the profile and general screening questionnaire is provided on the following pages.

APPENDIX B - continued

AIDS VACCINE EVALUATION GROUP
VOLUNTEER PROFILE

Date: ____________ Volunteer Number: ____________

Name: ____

Date of Birth: _____________ Social Security No. *[optional]*: _______________

Home Address: ____

____

Work Address: ____

____

Phone Number: (Day) _________________ (Evening) ________________

Can we contact you at home by phone? Yes/No ____ By mail? Yes/No ____

Can we contact you at work by phone? Yes/No ____ By mail? Yes/No ____

Name and address of person to contact in emergency:

____

____

____

Phone Number: (Day) ________________ (Evening) ________________

Relationship to you: ____________________________________

Name and address of an additional person to contact in case of an emergency:

____

____

____

Phone Number: (Day) ________________ (Evening) ________________

APPENDIX B - continued

AVEG GENERAL SCREENING QUESTIONS

Date: ______________________ *Volunteer Number:* __________

A. GENERAL HISTORY/SYSTEMS REVIEW *[Please answer **each** item]*

DO YOU HAVE NOW OR HAVE YOU HAD THE FOLLOWING:

	NO	YES [ever]	YES [in last 6 mos.]]	DON'T KNOW
Fever more than 2 weeks	☐	☐	☐	☐
Night sweats more than 2 weeks	☐	☐	☐	☐
Severe fatigue (tiredness)	☐	☐	☐	☐
Involuntary weight loss of more than 10 lbs.	☐	☐	☐	☐
Lymph node tenderness or enlargement	☐	☐	☐	☐
Anemia (low blood count)	☐	☐	☐	☐
Bruising or bleeding problem	☐	☐	☐	☐
Sinus infection (infection of the nose)	☐	☐	☐	☐
Gum or tooth problems	☐	☐	☐	☐
Mouth lesions (sores)	☐	☐	☐	☐
Skin rash or sores	☐	☐	☐	☐
Difficulty breathing	☐	☐	☐	☐
Asthma or wheezing	☐	☐	☐	☐
Positive PPD (TB skin test)	☐	☐	☐	☐
Tuberculosis	☐	☐	☐	☐
Pneumonia	☐	☐	☐	☐
Chest pain	☐	☐	☐	☐
Heart disease	☐	☐	☐	☐
High blood pressure	☐	☐	☐	☐
Loss of appetite	☐	☐	☐	☐
Stomach pain	☐	☐	☐	☐
Diarrhea	☐	☐	☐	☐
Ulcers	☐	☐	☐	☐
Yellow jaundice/hepatitis/liver disease	☐	☐	☐	☐
Gallbladder disease	☐	☐	☐	☐
Pain or difficulty with urination	☐	☐	☐	☐
Musculoskeletal pain/disease	☐	☐	☐	☐
Headache (more than 3 per month)	☐	☐	☐	☐
Memory loss or concentration difficulty	☐	☐	☐	☐
Visual (sight) changes	☐	☐	☐	☐
Hearing changes	☐	☐	☐	☐
Difficulty with walking, balance, coordination	☐	☐	☐	☐
Seizure	☐	☐	☐	☐
Depression (severe sadness)	☐	☐	☐	☐
Panic or anxiety disorder	☐	☐	☐	☐

APPENDIX B - continued

AVEG GENERAL SCREENING QUESTIONS

Date: ______________________ *Volunteer Number:* ____________

B. SPECIFIC MEDICAL QUESTIONS

		NO	YES	DON'T KNOW
1.	Have you ever been hospitalized?	☐	☐	☐
	If YES, give date and reason. ____			
2.	Has a doctor or other medical provider told you that you have a medical problem in the last 6 months?	☐	☐	☐
	If YES, specify with dates. ____			
3.	Are you currently taking any medicine?	☐	☐	☐
	If YES, specify what kind and for what reason. ____			
4.	Do you have any allergies or hypersensitivities?	☐	☐	☐
5.	Have you received blood/blood products transfusion within the past 6 months?	☐	☐	☐
6.	<u>Females only</u>:			
	Do you have menstrual problems?	☐	☐	☐
	Have you ever had an abnormal pap smear?	☐	☐	☐
	When was your last normal menstrual period? ____________________			
	Have you ever been pregnant?	☐	☐	☐
	How many pregnancies? _____			
	How many live births? _____			
	How many living children? _____			
	Have you ever had an abnormal breast exam or mammogram?	☐	☐	☐
7.	Have you ever been tested for HIV infection?	☐	☐	☐
	If YES, give approximate dates. ____			
8.	Do you plan to have a tattoo placed or skin pierced within the next year?	☐	☐	☐
9.	Are any of your household members less than 1 year old?	☐	☐	☐
10.	Do any have an immunodeficiency illness?	☐	☐	☐
11.	Do you plan to change residence within the next year?	☐	☐	☐

APPENDIX C

ALGORITHM FOR DETERMINATION OF HIV INFECTION

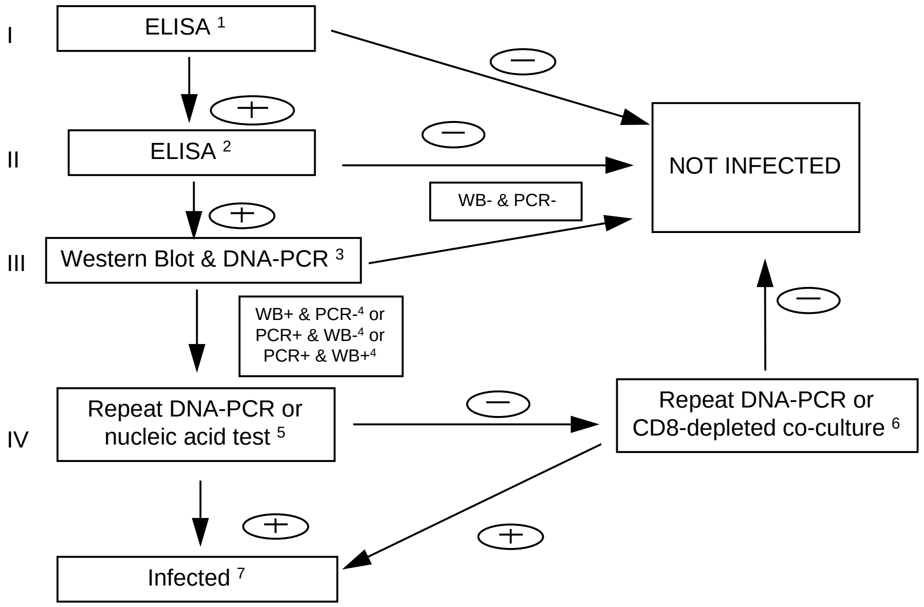

MAXIMUM TIME LINE

10 working days from volunteer visit

5 working days from I. positive

5 working days from II. positive

5 working days from III. positive

Note: Goal of 10-15 working days to complete Steps I-III

FOOTNOTES:

[1] Until a gp41 kit is approved by the Protocol 202 Steering Committee, the Abbott HIV-1,2 ELISA is used for both the first and second screening test. Upon approval, the gp41 kit will be substituted here.

[2] Abbott HIV-1,2 ELISA

[3] For procedure, use ACTG virology manual methodology, Roche Amplicor kit or a nested PCR technique.

[4] A Western Blot is only positive if declared such by the site immunologist. Western Blots with spurious or background bands, sometimes called “indeterminate”, should be considered <u>negative</u>. In the event of difficulty on calling a Western Blot + or -, the decision should be made to proceed to step IV.

[5] Consideration should be given to either using different PCR methodology, or to performing a CD8-depleted co-culture, but these are not required.

[6] If the ELISA and initial DNA-PCR are positive, and repeat DNA-PCR is negative, to absolutely exclude the possibility of a false negative, one further exam should be attempted.

[7] Once enrolled, confirmation should be attempted immediately by culture so as to isolate the circulating virus(es).

NOTES

A. The first ELISA and second ELISA can use the same specimen. The first ELISA could be run within 10 working days of receiving the sample. If a positive occurs, the follow-up ELISA must be set up within 5 working days.

B. The initial DNA-PCR and Western Blot should be performed from samples from the “A” time point above. These should be set up within 5 working days of a second positive ELISA.

C. If the DNA-PCR and WB trigger a confirmatory test, it should be run within 5 working days. These will require calling the volunteer back to the clinic to draw a new sample. The clinic coordinators should stress that we are running tests to help determine unclear results, and safe practices should be re-emphasized.

D. It is anticipated that the entire process will usually require about 15 working days. 25 working days will be the maximum acceptable time frame starting at the volunteer’s visit day.

APPENDIX D

EmulsiFlex 1000™
OPERATION AND CLEANING PROCEDURES

1.0 **PURPOSE**

To provide a procedure on the assembly/disassembly, operation and, cleaning of the Kirkland EmulsiFlex 1000™ used to facilitate preparation of an HIV-1 C4-V3 polyvalent peptide/ Montanide ISA-51 emulsion and placebo emulsion.

2.0 **SCOPE**

The EmulsiFlex 1000™ emulsifier, manufactured by Kirkland Products, is used to facilitate preparation of a peptide (in saline) and Freund's incomplete adjuvant emulsion prior to injection. The unit is constructed of stainless steel and has a hold up volume of less than 1 ml. O-rings are constructed of Viton.

The emulsion to be prepared is of a water-in-oil type. The “oil” portion of the emulsion will be provided by the Montanide ISA-51 product, while the “water” portion will be provided by either the peptide/saline mixture or saline alone.

A sample is forced through a low volume high pressure area and then past a valve where pressure is dropped and shear force occurs. The mixture continues around the valve and enters the second syringe. After each use, the unit will be flushed with hot (80ûC) 2% - 5% CIP-100 detergent solution and rinsed with Sterile Water for Injection (SWI). The unit will be sterilized at 121.1ûC for a minimum of 30 minutes before the next use.

3.0 **REFERENCE**

3.1 <u>EmulsiFlex 1000™ Technical Bulletin</u>

3.2 <u>Lederle-Praxis Biologicals Emulsiflex-SOP, dated December 10, 1996</u>

4.0 **PRECAUTIONARY MEASURES**

4.1 O-rings must be lubricated to prevent sticking and abrasion. Threaded areas can be lightly lubricated to facilitate turning. Lubrication will be accomplished by applying Montanide ISA-51® during the assembly process.

4.2 All assembly procedures must be performed in a sterile environment and utilizing sterile techniques in handling, including the use of sterile gloves.

4.3 Glass syringes must be used; oils (IFA) and solvents can soften and/or swell rubber plunger tips of disposable syringes.

APPENDIX D - continued

5.0 **MATERIALS AND EQUIPMENT**

5.1 EmulsiFlex 1000™

5.1.1 2 (two) sterile Emulsiflex 1000™ units. 1 (one) dedicated to HIV-1 C4-V3 polyvalent peptide/Montanide ISA-51 mixture and 1 (one) dedicated to Montanide ISA-51/saline mixture (placebo).

5.2 Clinical Materials

5.2.1 1 (one) HIV-1 C4-V3 polyvalent peptide vial [0.75 ml (750 μl)] containing 2 mg per 0.5 ml or 8 mg per 0.5 ml.

5.2.2 1 (one) vial containing sterile 0.9% NaCl for injection.

5.2.3 1 (one) 3.0 ml ampoule containing Montanide ISA-51.

5.2.4 1 (one) plastic tuberculin syringe with detachable 25 gauge 5/8Ó needle [Becton Dickinson and Co.]

5.2.5 2 (two) sterile 5 ml Luer-Lock glass syringes [Popper & Sons, Inc. MicroMate].

5.2.6 3 (three) sterile 1 ml Luer-Lock glass tuberculin syringes [Popper & Sons, Inc. MicroMate].

5.2.7 2 (two) sterile 20 gauge 1-1/2" needles [B-D cat#5176] with yellow hub.

5.2.8 2 (two) sterile 22 gauge 1-1/2" needle [B-D cat#5156] with black hub.

5.3 Cleaning Materials

5.3.1 2% - 5% CIP-100 Cleaning Solution (Calgon Vestal).

5.3.2 pH paper strips.

5.3.2 Heat Source (hotplate, microwave).

5.3.4 Polypropylene wash tray.

6.0 **PROCEDURES**

6.1 Assembly/Disassembly (Refer to figure in section 6.4)

6.1.1 All o-rings must be lightly lubricated with Montanide ISA-51 prior to assembly.

APPENDIX D - continued

6.1.2 Place bypass seal thumbscrew (A) into bypass channel (B). Turn bypass valve (A) clockwise, until bypass channel (B) is closed. Exert gentle pressure when passing o-rings by internal threads. Do not overtighten. To open bypass channel (B), turn valve (A) counterclockwise, about 2 turns.

6.1.3 Attach one end of spring (K) to valve (J). Attach other end of spring (K) to spring tension thumbscrew (L). Make sure connections are tight. Place L-K-J tension thumbscrew unit into orifice (I). Turn L-K-J tension thumbscrew unit clockwise, until closed. Exert gentle pressure when passing o-rings by internal threads. Do not overtighten. Turn L-K-J tension thumbscrew unit counterclockwise ½ turn (equals approximately 1000 psig necessary for emulsion processing).

6.1.4 Place thumb ring (H) with piston (G) into cylinder (E). Exert gentle pressure when passing o-rings by internal threads. Turn thumbscrew at (G-H) clockwise, tightening snugly around piston (G) to prevent leakage.

6.1.5 To disassemble, remove thumbscrews (A), (L-K-J) and (G-H) by turning counterclockwise. Exert gentle pressure when passing o-rings by internal threads. Separate (K) from (L) and (J).

6.2 <u>Emulsification</u>

6.2.1 Attach two 20 gauge yellow hubbed needles, via Luer-Lock, one to each 5 ml glass syringe.

6.2.2 Just prior to emulsification, remove one ampule Montanide ISA-51, one vial appropriate HIV-1 C4-V3 polyvalent peptide and/or one vial 0.9% NaCl for injection, and one ampoule Montanide ISA-51¨ from storage at 4 ^{o} C (products must be cold during emulsification process). Roll each vial along benchtop to mix contents. Lightly tap vials on benchtop to force material to bottom of vials.

6.2.3 **For peptide-containing emulsion:** Use plastic tuberculin syringe to pierce septum of 0.9% NaCl vial and withdraw 0.75 ml (750 µl) into syringe. Inject contents into HIV-1 C4-V3 polyvalent peptide vial; mix well. Discard tuberculin syringe. Use one 5 ml syringe to pierce septum of vial containing HIV peptide/NaCl mixture, and withdraw **entire** contents into syringe; note volume. Using the other 5 ml syringe, withdraw an equal volume of Montanide ISA-51. Express all air from both syringes.

6.2.4 **For placebo emulsion:** Use one 5 ml syringe to pierce septum of 0.9% NaCl vial, and withdraw 1.5 ml. Using the other 5 ml syringe, withdraw 1.5 ml of Montanide ISA-51. Express all air from both syringes.

6.2.5 Remove 20 gauge needles from syringes without any loss of contents (when holding syringes, hold both barrel and plunger to avoid any plunger motion which might eject contents, and to avoid sudden separation of plunger/syringe assembly, resulting in loss of contents).

APPENDIX D - continued

6.2.6 Attach HIV peptide/NaCl syringe via Luer-Lock to the EmulsiFlex 1000 at position C. With bypass valve A open, gently push HIV peptide/NaCl through channel until it appears at opening of position D. Attach Montanide ISA-51 syringe via Luer-Lock to the EmulsiFlex 1000 at position D. Introduce as little air as possible to the assembly.

6.2.7 Make sure connections are tight.

6.2.8 Slowly depress plunger of syringe at position C; majority of solution is now in syringe at position D. Wait approximately five seconds for the oil/water layers to separate. Once a clear boundary appears, slowly depress plunger of syringe at position D to drive materials back into syringe at position C. Again, wait for layers to separate. In this fashion, depress each plunger ten times, waiting at least three seconds between plunger depressions for materials to equilibrate.

6.2.9 Close bypass valve A with all material contained in syringe at position C.

6.2.10 Pull back on piston G, drawing material into channel E.

6.2.11 Push piston G in, forcing material into syringe D.

6.2.12 Repeat steps 6.2.9 and 6.2.10 until all material has been transferred from syringe at position C to syringe at position D.

6.2.13 Open bypass valve A; slowly depress plunger of syringe at position D, returning all contents to syringe at position C.

6.2.14 Close bypass valve A with all material contained in syringe at position C.

6.2.15 Remove original syringe at position D and attach 1 ml glass tuberculin syringe.

6.2.16 Repeat steps 6.2.9 and 6.2.10 until syringe at position D contains a minimum of 0.7 ml (0.5 ml per dose plus 0.2 ml for needle).

6.2.17 Repeat steps 6.2.14 and 6.2.15.

6.2.18 Remove syringe from Emulsiflex 1000 and attach 22 gauge black hubbed needles to both syringes for delivery to subject [2 (two) 0.5 ml doses for total dose of 1.0 ml per subject].

6.2.19 Attach third 1 ml glass syringe and repeat steps 6.2.9 and 6.2.10 until remaining emulsion has been collected.

6.2.20 Perform water drop test on remaining emulsion (third 1 ml glass syringe). Cover bottom of 60x15 mm petri dish with $4^{o}C$ water. Remove glass syringe containing remaining emulsion from Emulsiflex 1000 (refer to step 3 for warnings), and hold at 60° angle approximately 1 cm above surface of water. Let one drop fall onto surface of water. Drop should not disperse completely on water surface, and should remain largely entire for at least three minutes.

APPENDIX D - continued

6.3 Cleaning/Sterilization

6.3.1 Prepare 5% CIP-100 cleaning solution by adding 50 ml of CIP-100 concentrate to 950 ml sterile water. Heat solution to 80°C $\pm$ 10°C. (Microwave on high for 10 minutes).

6.3.2 While assembled, flush pre-mixing channel of Emulsiflex with hot 5% CIP-100 using either syringe C) or (D) to draw up cleaning solution. Repeat until no detection of emulsion is visible in either syringe using fresh 5% CIP-100 in between flushes (approximately seven times).

6.3.3 Flush final-processing channel of Emulsiflex 1000 with hot 5% CIP-100 using syringe C) to draw up fresh cleaning solution. Repeat until no detection of emulsion is visible in either syringe using fresh 5% CIP-100 in between flushes (approximately seven times).

6.3.4 Disassemble. Wipe excess emulsion off each component with Kim-wipes. Place into polyproylene tray. Overpour with remaining hot 5% CIP-100. Let soak for 15 minutes.

6.3.5 Rinse with deionized water until neutral pH. Assemble as described in Section 6.1.

6.3.6 Repeat steps 6.3.1 through 6.3.3. (Flush a minimum of two times.)

6.3.7 Disassemble. Place into polypropylene tray. Overpour with remaining hot 5% CIP-100. Let soak 15 minutes.

6.3.8 Rinse with deionized water until neutral pH.

6.3.9 Flush pre-mixing channel with fresh deionized water until neutral pH, then flush final-processing channel with fresh deionized water until neutral pH.

6.3.10 Unless autoclaving within 24 hours, dry inner channels using compressed air/nitrogen.

6.3.11 Place each component into sterilization pouch. Sterilize at 121.1°C (minimum) for minimum of 30 minutes with sufficient drying time.

APPENDIX D - continued

6.4 Kirkland Emulsifier Diagram

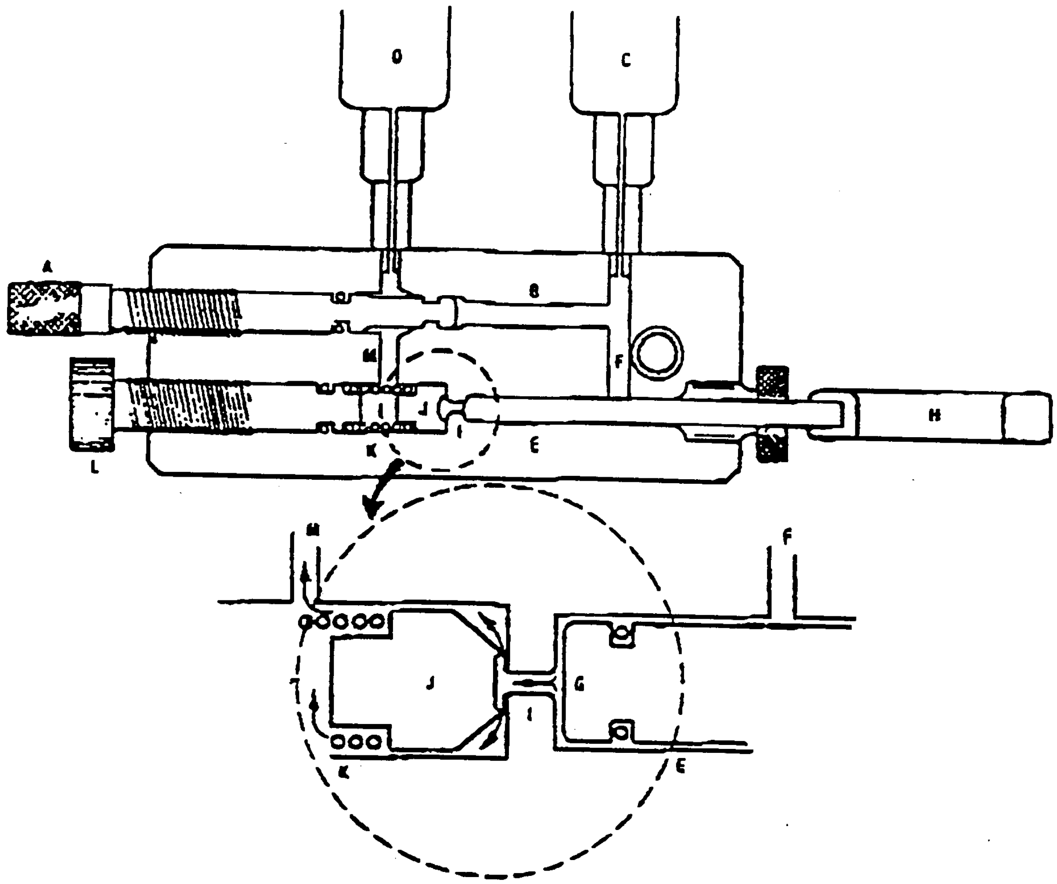

APPENDIX E - **REVISED**

TIME COURSE FOR IMMUNOGENICITY DETERMINATIONS

[Refer to Sections 10.2 and 10.3 for details]

**Month**			0	1		2	3	6		7	10	12		13	15	18
**DAY**		-56 to -1	0	28	42	56	112	182	196	210	294	364	378	392	448	532
**STUDY VISIT**		**01**	**02**	**03**	**04**	**05**	**06**	**07**	**08**	**09**	**10**	**11**	**12**	**13**	**14**	**15**
Immunization			X	X												
**HUMORAL**	**SITE**															
HIV ELISA	AVEU	X											X			
Western Blot	AVEU	[X]											X			
ELISA panel	CIL	X		X	X	X			X				X			
CD4 binding inhibition[1]<br>Fusion inhibition[1]	CIL								X				X			
Neutralizing antibody[1]	CIL								X				X			
**CELLULAR**																
CTL, LDA[2], and other ancillary cellular assays	CIL;<br>AVEU/BI		X		X				X				X			
LPA	VAN	X		X		X			X[3]				X[3]			

NOTES: Assays will be done on all volunteers, unless indicated otherwise [ ] If indicated

Shading indicates visits that were required in the original protocol, but are no longer required

[1] Heterologous if homologous positive

[2] See text for details

[3] Previously required at Days 182 and Day 364, respectively

APPENDIX F - **REVISED**

CALENDAR OF STUDIES

**Month**		0	1		2	3	6		7	10	12		13	15	18
**DAY**	-56 TO -1	0	28	42	56	112	182	196	210	294	364	378	392	448	532
**VISIT**	**01**[1]	**02**	**03**	**04**	**05**	**06**	**07**[1]	**08**	**09**	**10**	**11**[1]	**12**	**13**	**14**	**15**[1]
Immunization		X	X[4]												
History and Physical Exam	X											X†[6]			
Clinical Evaluation		X[1]	X[1]	X	X			X							
Urine Dipstick	X		X	X				X				X			
Pregnancy Test	X	[X]	X[4]									X[7]			
HBsAg; RPR; PPD; chest x-ray	X														
ALT, GGT, Creatinine	5		5	5				5				5			
CBC, Differential, Platelets	5		5	5				5				5			
T Cell Subsets	7		7	7				7				7			
Heparinized blood : EBV[1]	40														
Heparinized blood: HLA	15														
ACD blood: CTL,LDA[1] and other ancillary studies		30-70		70				70				70			
ACD blood: LPA; cryopreservation	120		90		90			90[5]				90[5]			
DTH[2]												X[6]			
Serum for Antibody Studies; Storage; Distribution	50[3]		50	50	50	50		50				50			

NOTES: <u>Shading indicates visits that were required in the original protocol but are no longer required</u> [ ] if indicated † Social Impact Survey

1 See text for details
2 Placed at Day 378; read at Day 380
3 HIV serology required
4 Required only for a subset of volunteers; see text for details
5 Previously required at Days 182 and 364, respectively
6 Previously required at Day 532
7 Chest x-ray only
